# When pitch adds to volume: coregulation of transcript diversity predicts gene function

**DOI:** 10.1186/s12864-018-5263-z

**Published:** 2018-12-13

**Authors:** Alejandro Cáceres, Juan R. González

**Affiliations:** 1 ISGlobal, 08003 Barcelona, Spain; 2 Centro de Investigación Biomédica en Red en Epidemiología y Salud Pública (CIBERESP), Madrid, Spain; 3 Department of Mathematics, Universitat Autònoma de Barcelona, 08193 Bellaterra (Barcelona), Spain

**Keywords:** Alternative splicing, Transcription diversity, Co-splicing, Transcriptome, Epistasis, Alzheimer’s disease, Gene function prediction, RNA-sequencing

## Abstract

**Background:**

Genes corregulate their overall transcript volumes to perform their physiological functions. However, it is unknown if they additionally coregulate their transcript diversities. We studied the reliability, consistency and functional associations of co-splicing correlations of genes of interest, across two independent studies, multiple tissues and two statistical methods. We thoroughly investigated the reproducibility of co-splicing correlations of *APP*, the candidate gene of Azheimer’s disease (AD). We then studied how co-splicing correlations in different tissues contributed to predict functional interactions of three other genes and finally computed co-splicing frequency for 17 thousand genes across 52 human tissues.

**Results:**

We replicated co-splicing correlations between *APP* and 5 AD-related genes and reproduced expected enrichment of *APP* co-splicing in synaptic vesicle cycle and proteosome pathways. We observed novel associations for tissue vulnerability to disease with enrichment in *APP* co-splicing, co-expression and epistasis in AD. *APP* co-splicing was the strongest predictor and replicated between studies. We confirmed known gene interactions of *PRPF8* and *GRIA1* in testis and brain cortex, and observed a novel interaction of *FGFR2*, in breast and prostate, modulated by cancer risk-variants. We produced a co-splicing map across 52 human tissues to help predict the function of over 17 thousand genes.

**Conclusions:**

We show that coregulation of transcript diversities provides novel biological insights in gene physiology and helps to interpret GWAS results. Co-splicing correlations are reliable and frequent and should be further pursued to help predict gene function. Our results additionally support current AD interventions aiming at the ubiquitin proteosome pathway but unveil the need to consider transcript diversity in addition to volume to assess treatment response and susceptibility to the disease.

**Electronic supplementary material:**

The online version of this article (10.1186/s12864-018-5263-z) contains supplementary material, which is available to authorized users.

## Background

The correlation between gene expression levels has been a prominent tool to study the emergence and conservation of biological functions [1]. However, it is now clear that most genes support a diverse repertoire of transcript isoforms [2], produced by alternative transcription initiation, alternative polyadenylation and alternative splicing [3]. Consequently, gene expression can change not only in volume but also in quality when some isoform groups are more frequently produced than others. As coregulation of gene expression contributes to orchestrate the function of genes, it is then conceivable that genes may also tune in their transcript distributions in functions that involve selected isoforms.

The transcript diversity of genes is strongly dependent on the tissue [4], suggesting ample physiological consequences of its regulation [5]. Some insights have been made from functional associations of gene coregulation networks. Recently, Iancu and colleagues described co-splicing correlations across species as a measure of the coregulation of the transcript diversities between gene pairs [6]. They observed that network hubs strongly represented neurobiological functional pathways. More recently, Saha et al. demonstrated strong presence of splicing regulatory hubs from co-splicing networks across numerous tissues, using Genotype Tissue-Expression (GTEx) data [7]. To gain further understanding into the biological consequences of the regulation of transcript diversity, we ask whether significant coregulation among genes is consistent with their physiological functions. Therefore, unlike previous studies that aimed to identify structural properties of co-splicing networks, here, we considered whether the co-splicing correlations of a gene of interest play a relevant role in the gene’s biological function. In this context, we attempted to assess how reliable, widespread and informative on gene function co-splicing correlations are.

We therefore aim to first study the reproducibility and biological significance of co-splicing correlations of a candidate gene for which there is evidence that splicing ratios play an important role. *APP* is a prominent candidate gene for Alzheimer’s disease (AD) [8]. The gene supports numerous isoforms [9] and research indicates that its spliceform ratios are different between AD patients and controls, triggering the amyloid cascade and apoptosis or being involved in aging hippocampi [10–13]. In addition to the hypothesized toxicity of APP’s amyloid-β fragment, disruptions of the gene’s function may also be important contributors to the disease [14]_._ Increasing our understanding of *APP*’s physiological regulation is, therefore, essential. Validating between independent studies, methods and brain regions, we therefore aimed to determine the robustness of *APP* co-splicing correlations with genes and pathways that contribute to the gene’s physiological function and role in AD. We therefore analyzed two independent studies (GTEx and BRAINEAC) [15, 16], two co-splicing methods and ten different brain regions to fully characterize the transcriptome-wide co-splicing of *APP*. To assess the further biological insight given by the regulation of transcription diversity, we studied the relative contribution of *APP* co-splicing, co-expression and genome-wide epistasis (defined in Methods) to a transcriptomic signature of tissue vulnerability for the disease [17].

Since, to our knowledge, this is the first effort to try to infer a gene’s function through transcriptome-wide coregulation of transcript diversity, we then studied whether transcriptome-wide co-splicing correlations were also relevant to predict the function of other genes in different tissues. We therefore hypothesized that transcriptome-wide co-splicing of genes of interest in relevant tissues predicts expected protein-protein interactions and informs on plausible new interactions. We thus studied *PRPF8, GRIA1* and *FGFR2*, which are involved in the regulation of splicing in testis, glutamatergic neurotransmission in brain cortex and susceptibility to breast cancer, respectively [18–21]. Finally, we assessed the frequency of co-splicing correlations across 17 thousand genes and 52 human tissues and made the results available at coSplicing4GTEx web application as novel gene function predictor [22].

## Results

### Methods to compute co-splicing correlations

**Fig. 1:**
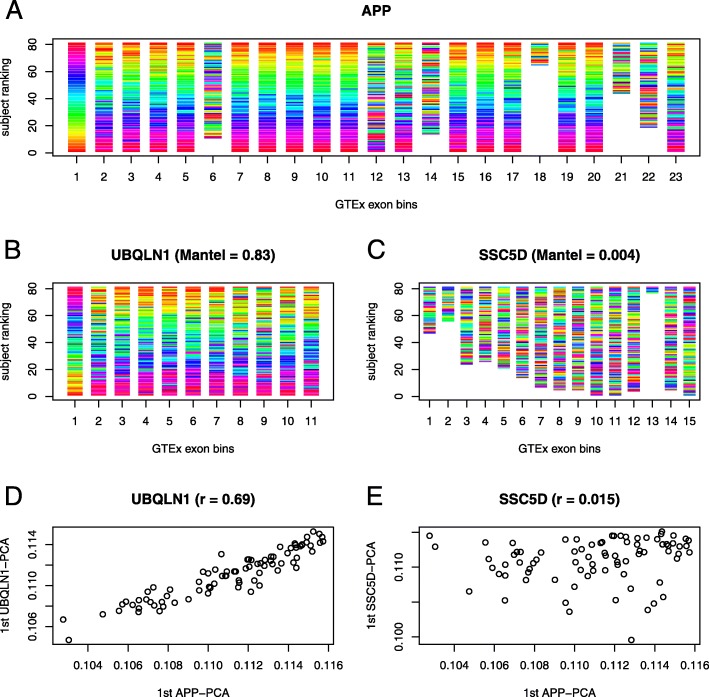
Co-splicing correlations of *APP* with *UBQLN1* and *SSC5D* in hippocampus (GTEx). **a** Equalizer type plot displaying the exon count distributions of *APP* ranked across individuals at each exon, with maximum possible ranking for tie. Colors follow the ranking of exon 1 of *APP.* **b** The subject ranking of exon count distributions of *UBQLN1,* colored by the ranking of *APP* exon 1, shows a clear coherence between **a** and **b**. The tuning of exon count distributions between genes leads to similar distance matrices between individuals and results in high Mantel’s correlations. **c** For *SSC5D* the ranking coherence is broken, Mantel’s correlation is therefore low and there is no co-splicing with *APP*. **d** The first principal component (PC), across individuals, of the exon count distributions of *APP* highly correlates with that of *UBQLN1,* adjusting for covariates. **e** The first PC of *APP* does not correlate with the first PC of *SSC5D*, suggesting low splicing coregulation

Gene expression levels are intensively used to determine groups of genes that by coregulating their transcription volumes perform biological functions. However, it is conceivable that genes also coregulate their transcription diversities to determine which transcript isoforms should be involved in a particular function. The coregulation of transcript diversity has been recently estimated from co-splicing correlations [6, 7]. From RNA-seq data, co-splicing correlations across subjects are derived from the exon count distributions of genes [6, 7]. As defined by Iancu and colleagues, the co-splicing between two genes can be computed from the Mantel’s correlation between the distance matrices of exon count distributions across subjects [6] (see Methods). We propose to visualize these co-splicing correlations back in terms of exon count data as shown in Fig. 1**.** The figure illustrates two genes in high co-splicing, *APP* and *UBQLN1, and low co-splicing, APP and SSC5D*, derived from the exon count data (RNA-seq) of hippocampus from the GTEx project, version 6. High co-splicing between two genes, as measured by Mantel’s correlation, results in a coherent ranking of individuals across numerous exons between genes. We thus observe that the subject ranking of count frequencies at *APP*’s exon 1 is coherent with the rankings across numerous exons of *APP* and *UBQLN1* but not across the exons of *SSC5D* (compare Fig. 1a with b and with c). To gain further insight into the coregulation of transcription diversity, we additionally propose an alternative measure of co-splicing based on the first principal component (PC) of the exon count distributions across individuals. Interpreting the first PC as providing main differences between transcript mixtures between individuals for a given gene, co-splicing can then be measured as the correlation between the first PCs of two genes. PC-based co-splicing is computed by the partial correlation adjusting for covariates. We observed, in the example of Fig. 1, that the partial correlation between the first PCs of genes is high between *APP* and *UBQLN1* and low between *APP* and *SSC5D* (Fig. 1d and e). The PC based method was used for validation of Mantel’s correlations and to test interactions with genomic variants.

### Co-splicing complements co-expression

**Fig. 2:**
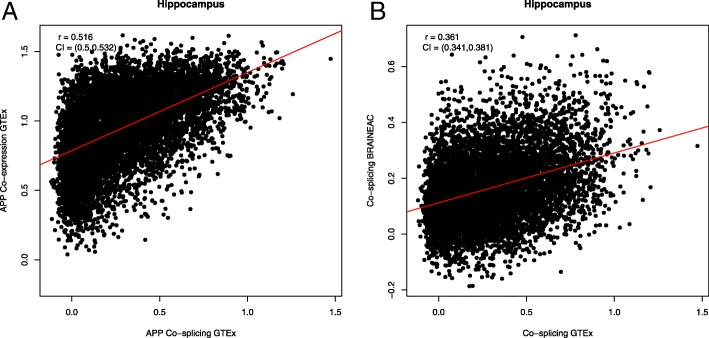
Consistency and reproducibility of *APP* co-splicing in hippocampus. **a** Correlation between transcriptome-wide *APP* co-expression and co-splicing correlations. Each point is a gene for which its co-expression and co-splicing correlations (*z*-transformed) with *APP* were computed. A high correlation was found for hippocampus data of GTEx, where high co-splicing is a clear subset of high co-expression: {genes such that *z* co-splicing > 1} ⊂ {genes such that *z* co-expression > 1}, where *z* = 1 corresponds to a correlation of 0.76. **b** Reproducibility between independent studies. Mantel co-splicing correlations (z-transformed) with *APP* was computed in two independent studies (GTEx and BRAINEAC). While the correlation is moderate, it is highly significant, showing that genes with high *APP* co-splicing in GTEx are also likely to be high in *APP* co-splicing in BRAINEAC. Co-splicing correlations in GTEx are higher than in BRAINEAC

We aimed to characterize the co-splicing of *APP* from its relationship to co-expression. We first studied the association between transcriptome-wide co-expression and co-splicing of *APP* with 17,368 genes in the hippocampus data of GTEx (*N* = 94), one of the main regions affected in AD. We computed the transcriptome-wide co-expression of *APP* using a partial Pearson’s correlations of kilobase per million mapped reads (RPKM), adjusting by tissue-specific covariates. We estimated transcriptome-wide co-splicing from Mantel’s correlations, also adjusting by covariates. We noticed high correlation between *APP* co-expression and co-splicing (Pearson’s *r* = 0.51, *P* < 1 × 10^− 16^, Fig. 2a). Interestingly, genes in high co-splicing with *APP* appear as a clear subset of those in high co-expression, indicating that coregulation of transcript diversity is likely accompanied by coregulation of transcription volume; as some isoforms become more abundant, they increase the overall transcription of genes. Whereas, changes in trascription levels do not necessarily imply changes in splicing ratios.

### Reliability of co-splicing correlations between different methods, studies and tissues

We studied the replication of co-splicing of *APP* with different inference methods and its validation across two independent studies and numerous brain regions. Using exon-array data of hippocampus from the BRAINEAC project (*N* = 130), we investigated the reproducibility of transcriptome-wide co-splicing of *APP* between studies and inference methods. We computed, in both studies, the co-splicing correlations of *APP* with 12,577 commonly-annotated genes, using the Mantel and PC methods, to determine the level of agreement between analyses and studies. We found high agreement with a Pearson’s correlation of 0.71 and 0.52 between methods, in GTEx and BRAINEAC, respectively (Additional file 1: Figures S1-S2). We also calculated the consistency of the transcriptome-wide co-splicing of *APP* between studies (Fig. 2b). While the correlation between studies was moderate (0.36), it was highly significant (*P* < 1 × 10^− 16^), demonstrating that a high degree of *APP* co-splicing across the genome was reproduced between studies. This is a relevant result, as the differences between studies are numerous: particularly, microarray’s exon expression distributions are reconstructed from subject ranking (see Methods); and more generally, they differ in samples, experiments (RNAseq and microarray), data reduction algorithms and co-splicing inference.

We then studied the co-splicing of *APP* across multiple brain regions, where deposition of amyloid plaques begins [23]. We considered six different brain regions from GTEx and four form BRAINEAC (see Methods), and searched for the genes that were significantly co-spliced with *APP* across methods, brain regions and studies. We observed, in particular, that the PC-based co-splicing method in hippocampus had more statistical power than Mantel’s correlation; *P*-values for Mantel’s correlations were computed from the normal distribution of z-transformed correlations, further standardized and cube-root transformed to account for right skewness (Additional file 1: Figures S3-S4). Genes with significant co-splicing corrected for multiple comparisons were identified from the PC-based method and validation between studies was based on Mantel’s correlations, the reference method. As a result, a total of 11 genes with reproducible co-splicing across methods, regions and studies were identified, see Table 1. Five genes, *UBQLN1, APLP2*, *ATP2A2, ATP6AP2* and *DNER*, have been previously linked to AD, suggesting probable disruptions of *APP*’s physiological regulation during disease. *UBQLN1* links proteosomes and ubiquitin ligases for protein degradation and its variants have been associated to AD [24], *APLP2* has been involved in glutamatergic transmission and synaptogenesis in association with *APP* [25, 26], *ATP2A2* is a SERCA Ca(2+) ATPase, involved in calcium homeostasis in the endoplasmatic reticulum, and regulates amyloid-β production [27], *ATP6AP2* is a constituent of the renin-angiotensin system whose down-regulation has been associated to AD [28] and *DNER* promotes glia differentiation activating gamma secretase signaling [29].

**Table 1:** Genes with significant *APP* co-splicing between studies (GTEx and BRAINEAC) and different brain regions (HIPP: hippocampus, AMYG: amygdata, HYP: hypotalamus, ACTX: anterior cingulate cortex, CTX: cortex, FCTX: frontal cortex, OCTX: occipital cortex, TCTX: temporal cortex)

Genes	GTEx							BRAINEAC			
	HIPP *PCA-cor (P)*	HIPP *M-cor (P)*	AMYG *M-cor (P)*	HYP *M-cor (P)*	ACTX *M-cor (P)*	CTX *M-cor (P)*	FCTX *M-cor (P)*	HIPP *M-cor (P)*	FCTX *M-cor (P)*	OCTX *M-cor (P)*	TCTX *M-cor (P)*
*DNAJC6*	0.75 (1.1e-13)	0.83 (0.007)	0.84 (0.002)	0.84 (0.02)	0.89 (0.004)	0.79 (0.003)	0.91 (0.009)	0.52 (0.002)	0.32 (0.013)	0.42 (0.006)	0.3 (0.038)
*DNER*	0.71 (9.0e-12)	0.75 (0.019)	0.71 (0.014)	0.85 (0.017)	0.81 (0.014)	0.65 (0.018)	0.8 (0.039)	0.38 (0.022)	0.31 (0.015)	0.34 (0.027)	0.35 (0.019)
*UBQLN1*	0.69 (6.6e-11)	0.83 (0.007)	0.73 (0.01)	0.82 (0.026)	0.75 (0.027)	0.72 (0.008)	0.8 (0.039)	0.52 (0.002)	0.52 (1e-4)	0.44 (0.004)	0.48 (0.002)
*ATP6AP2*	0.68 (9.0e-11)	0.82 (0.008)	0.82 (0.003)	0.86 (0.015)	0.87 (0.006)	0.75 (0.006)	0.92 (0.008)	0.48 (0.004)	0.35 (0.007)	0.43 (0.005)	0.32 (0.028)
*APLP2*	0.68 (1.9e-10)	0.77 (0.016)	0.59 (0.047)	0.75 (0.048)	0.76 (0.025)	0.67 (0.014)	0.81 (0.037)	0.42 (0.012)	0.39 (0.003)	0.42 (0.006)	0.43 (0.004)
*TPD52*	0.66 (6.0e-10)	0.79 (0.013)	0.75 (0.008)	0.81 (0.028)	0.82 (0.012)	0.7 (0.01)	0.9 (0.011)	0.4 (0.018)	0.32 (0.013)	0.43 (0.005)	0.36 (0.016)
*PRKAR1A*	0.64 (3.3e-09)	0.8 (0.011)	0.81 (0.003)	0.78 (0.038)	0.8 (0.016)	0.72 (0.008)	0.83 (0.03)	0.41 (0.015)	0.29 (0.022)	0.34 (0.029)	0.36 (0.014)
*ACLY*	0.63 (5.1e-09)	0.79 (0.013)	0.7 (0.016)	0.79 (0.035)	0.88 (0.004)	0.73 (0.006)	0.89 (0.013)	0.5 (0.003)	0.31 (0.017)	0.46 (0.002)	0.4 (0.007)
*ATP2A2*	0.58 (2.0e-07)	0.81 (0.009)	0.77 (0.006)	0.77 (0.042)	0.8 (0.016)	0.8 (0.002)	0.82 (0.031)	0.5 (0.003)	0.37 (0.004)	0.35 (0.021)	0.37 (0.012)
*STAU1*	0.56 (6.5e-07)	0.77 (0.016)	0.7 (0.016)	0.79 (0.033)	0.8 (0.015)	0.69 (0.011)	0.81 (0.037)	0.41 (0.015)	0.44 (0.001)	0.44 (0.004)	0.44 (0.004)
*MTMR4*	0.51 (6.2e-06)	0.74 (0.022)	0.64 (0.029)	0.77 (0.042)	0.81 (0.014)	0.62 (0.023)	0.87 (0.018)	0.41 (0.016)	0.34 (0.008)	0.35 (0.021)	0.32 (0.029)

The first column shows the co-splicing correlations obtained by the PC method and corresponding *P*-values in parenthesis. Mantel’s correlations follow from the second column

### Modulation of co-splicing by a SNP

Notably, we found reproducible *APP* co-splicing with *UBQLN1,* which in physiological state is a chaperone of *APP* that contributes to its synthesis and processing [30]_._ *UBQLN1’*s alternative splicing and common variation has been associated with increased susceptibility to AD [24, 31–33], not without controversy [34, 35]_._ Since the polymorphism UBQ-8i (rs12344615) in *UBQLN1* has been associated with increased risk of late-onset Alzheimer’s disease (LOAD) and differences in *UBQLN1* exon 8 splicing*,* we asked whether *APP/UBQLN1* co-splicing was modulated by UBQ-8i. We found that, in the frontal cortex, the interaction between the first PC of *APP*’s exon count distribution and the SNP was significantly associated with the first PC of *UBQLN1* (β = − 0.08, *P* = 0.035, Additional file 1: Figure S5). We adjusted for 6 genome-wide surrogate covariates and sex. While the interaction was not significant in other tissues tested, they all showed a consistent direction of the estimate, suggesting a link between UBQ-8i and *APP* splicing ratios.

### Contribution of *APP*’s co-splicing, co-expression and epistasis in tissue vulnerability to AD

We then studied whether transcriptome-wide co-splicing of *APP* lead to significant enrichment of biochemical pathways, exploring further the physiological functions of *APP* at pathway level through co-splicing correlations. We computed the pathway enrichment of *APP* co-splicing on 277 KEGG pathways, given by genes with *APP* co-splicing *P*-value > 0.05. The overall agreement between the GTEx and BRAINAC studies to declare a pathway significantly enriched in *APP* co-splicing was given by a Cohen’s κ of 0.37 (*P* = 1.71 × 10^− 10^), in line with the observed reproducibility at gene level. After correcting for multiple comparisons, that included number of pathways and brain regions, we observed a total of six significant pathways validated between studies (Table 2). Consistent with known *APP*’s physiological functions, S*ynaptic vesicle cycle* showed the most significant combined *P*-value between experiments [36] (*P* = 8.67 × 10^− 7^)_,_ followed by *Protein processing in endoplasmic reticulum* [37] (*P* = 2.10 × 10^− 5^), a pathway in which *UBQLN1* is involved (Additional file 2: Table S1). Also relevant to AD, *Ubiquitin mediated proteolysis* was reproducible between studies, as it was *long term potentiation.* Consistent with co-splicing being a factor influencing co-expression, we confirmed that all pathways with reproducible enrichment in co-splicing were also enriched in co-expression and that many more pathways were enriched in co-expression than co-splicing (Additional file 2: Table S1).

**Table 2:** *KEGG pathways enriched in co-splicing with APP in GTEx and BRAINEAC, across limbic and neocortex regions*

STUDY	KEGG	Pathway Description	corrected *P*
GTEx	hsa04070	Phosphatidylinositol signaling system	8.93E-4
	hsa04728	Dopaminergic synapse	8.93E-4
	**hsa04141**	**Protein processing in endoplasmic reticulum**	**4.04E-3**
	**hsa04120**	**Ubiquitin mediated proteolysis**	**5.32E-3**
	hsa04022	cGMP-PKG signaling pathway	6.82E-3
	hsa04730	Long-term depression	1.46E-2
	hsa04530	Tight junction	1.65E-2
	hsa04724	Glutamatergic synapse	1.67E-2
	hsa04520	Adherens junction	1.80E-2
	hsa04713	Circadian entrainment	2.28E-2
	hsa04723	Retrograde endocannabinoid signaling	2.36E-2
	**hsa04261**	**Adrenergic signaling in cardiomyocytes**	**3.18E-2**
	**hsa04721**	**Synaptic vesicle cycle**	**3.34E-2**
	hsa04727	GABAergic synapse	3.34E-2
	hsa05130	Pathogenic *Escherichia coli* infection	3.47E-2
	**hsa04114**	**Oocyte meiosis**	**4.75E-2**
	hsa04611	Platelet activation	4.75E-2
	hsa04015	Rap1 signaling pathway	4.75E-2
	**hsa04720**	**Long-term potentiation**	**4.94E-2**
BRAINEAC	hsa4142	Lysosome	1.82E-9
	**hsa4721**	**Synaptic vesicle cycle**	**1.46E-6**
	hsa4961	Endocrine and other factor-regulated calcium reabsorption	6.20E-6
	**hsa4141**	**Protein processing in endoplasmic reticulum**	**3.62E-4**
	hsa5110	Vibrio cholerae infection	8.07E-4
	hsa4144	Endocytosis	1.04E-3
	**hsa4261**	**Adrenergic signaling in cardiomyocytes**	**2.65E-3**
	**hsa4114**	**Oocyte meiosis**	**1.00E-2**
	**hsa4720**	**Long-term potentiation**	**1.00E-2**
	hsa4145	Phagosome	1.20E-2
	**hsa4120**	**Ubiquitin mediated proteolysis**	**1.28E-2**

*P*-values are corrected for the number of pathways tested (277) and brain regions. Pathways in bold face were validated between studies

**Fig. 3:**
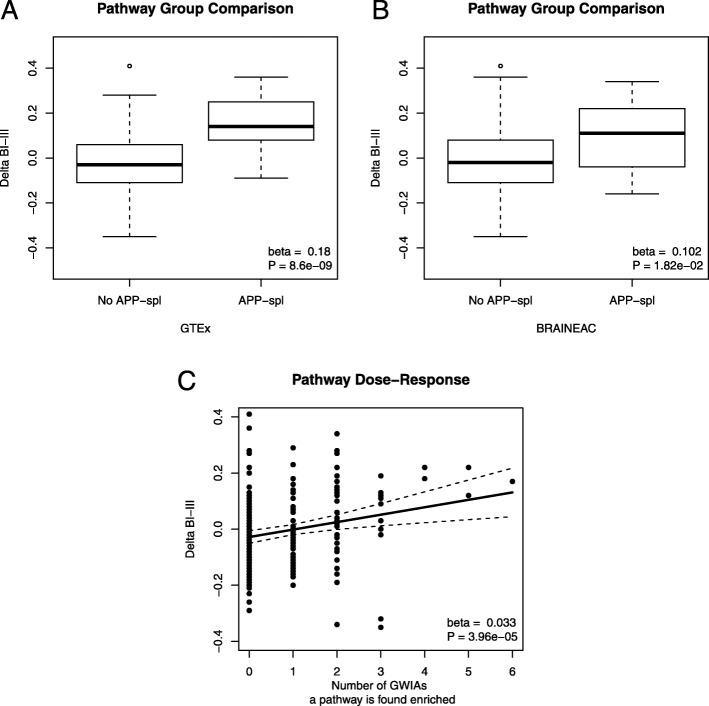
Association between the tissue vulnerability score of AD (Delta BI-III) and enrichment of *APP* co-splicing, and multiple enrichment in *APP* epistasis. **a** Box plots (box: 3rd, 2nd and 1st quartiles, whiskers: outlier fences of 1.5 interquartile range) representing the distributions of Delta BI-III for pathways with no significant enrichment in *APP* co-splicing (No *APP*-spl) and for pathways with significant enrichment (*APP*-spl), for GTEx data**.** The difference between means (beta) and *P*-value are reported at the bottom. **b** Similar plot to (a) for BRAINEAC data. **c** Dose-response relationship between tissue vulnerability and multiple enrichment in *APP* epistasis. Each dot is a pathway. The x axis shows the number of genome-wide interaction analyses (GWIAs), from 27 SNPs in *APP*, in which a pathway was found significantly enriched

To determine a putative impact of a deregulation of transcript diversity in disease, we correlated the pathway’s enrichment in *APP* co-splicing with a recent transcriptional signature of tissue vulnerability to AD [17] The signature is based on expression differences between Braak regions I to III and unaffected brain regions, and it is enriched for pathways that co-aggregate with amyloid-β and tau protein, in plaques and tangles [17]. As a measure between normal *APP* functioning and genetic susceptibility to disease derived from inadequate protein degradation, we tested whether the pathway vulnerability score correlated with enrichment in *APP* co-splicing (Additional file 2: Table S1). We observed that the tissue vulnerability score of pathways strongly correlated with their enrichment in *APP* co-splicing, both in GTEx (*β* = 0.18, *P* = 8.6 × 10^− 9^) and BRAINEAC (*β* = 0.10, *P* = 0.018, Fig. 3a and b). We also found that enrichment in co-expression correlated with the vulnerability score weaker than co-splicing and was not validated between studies (*β* = 0.08, *P* = 1.1 × 10^− 4^ in GTEx, *β* = 0.05, *P* = 0.13 in BRAINEAC). This results shows that the specificity gained by enrichment in co-splicing improved the associations between the physiological function of *APP* and the vulnerability score.

We applied an enrichment analysis of epistatic effects as described in Caceres et al. [38], with the objective of finding significant genomic associations of co-splicing correlations at a pathway level. Specifically, we asked which pathways were enriched in interactions with multiple SNPs in *APP*, and if the pathways correlated with those enriched in *APP* co-splicing. This analysis is well powered (80%) to detect interactions with SNPs having odds ratios greater than 1.28 in studies with more than 1000 subjects, and false positive rate is kept under control when adjusting for multiple comparisons for the number of pathways tested **(**Additional file 1: Figures S6-S11). We analyzed three LOAD GWAS: ADG (2686/935 cases/controls), NIA (587/289) and GENADA (806/782). We pruned SNPs within *APP* keeping those that were in pair-wise linkage equilibrium at R^2^ < 0.2 within studies. We performed 27 genome-wide interaction analyses (GWIAs) for the 27 SNPs in *APP*, covered in the three studies (Additional file 1: Tables S2-S4). Each GWIA tested the interactions of one the SNPs in *APP* with all the SNPs genotyped across the genome. For each GWIA, we then performed enrichment analysis on 277 KEGG pathways. A pathway’s multiple enrichment in *APP* epistasis was defined as the total number of GWIAs in which the pathway was found significantly enriched (Additional file 3: Table S5). We did not find statistical evidence for the association between multiple enrichment in *APP* epistasis and enrichment in *APP* co-splicing (β = 0.18, *P* = 0.36 in GTEx, β = 0.13, *P* = 0.62 in BRAINEAC). However, we observed a strong correlation between multiple enrichment in *APP* epistasis and the tissue vulnerability score (robust regression, *β* = 0.033, *P* = 3.96 × 10^− 5^, *R*^*2*^ = 0.045, Fig. 3c), confirming a strong link between the vulnerability score and the genetic variability of *APP*. We fitted, in GTEx, a regression model for the vulnerability score that included enrichment of *APP* co-splicing, co-expression and epistasis, and observed that all the factors contributed significantly to the model (*F(3,208)* = 19.56, *P* = 3.2 × 10^− 11^, *R*^*2*^ = 0.22), being co-splicing the strongest contributor of all (*β* = 0.15, *P* = 2.7 × 10^− 7^).

### Co-splicing correlations predict gene function of *PRPF8* and *GRIA1* in testis and brain

We studied two additional genes of interest for which alternative splicing have shown to be relevant for the genes’ function. We first considered *PRPF8.* Prp8p is a component of the U5 assembly within the catalytic center of the spliciosome [18]. As testis is a tissue with high levels of splicing events, we looked at the genes that co-spliced the strongest with *PRPF8* in this tissue. Remarkably, in the top 20 genes with highest co-splicing, we observed *SNRNP200* (small nuclear ribonuclear protein U5 subunit 200), *SF3B3* (splicing factor 3B subunit 3), *PRPF6* (Pre-mRNA-processing factor 6) and *SF3B2* (splicing factor 3B subunit 2) with Mantel’s correlations of 0.9, 0.87, 0.86 and 0.85, respectively. Enrichment analysis of KEGG pathways confirmed *spliceosome* as the most significant pathway (adjusted-*P* = 1.24 × 10^− 4^). These findings suggest that the complex cascade of spliceosome interactions may be a physiological feature of alternative splicing beyond developmental stages [39] and are in line with the enrichment in splicing genes for the transcriptome-wide networks that combine expression and relative isoform levels [5].

We further looked at the transcriptome-wide co-splicing of *GRIA1* (Glutamate Receptor 1) in the brain cortex. Different isoforms of *GRIA1*–*4* combine in various tetramers to produce different versions of AMPA receptors for glutamate, each with a specific function [19]. We found that the most co-spliced gene in the genome with *GRIA1* was *GRIA3* (Mantel’s correlation 0.85). Interestingly *NSF,* which binds to AMPA receptor GluR2 subunit (*GRIA2*), was the second highest in co-splicing with *GRIA1* (0.84). From the top 20 genes in co-splicing with *GRIA1,* 4 were part of *synaptic vesicle cycle* (adjusted-*P* = 5.85 × 10^− 4^) and 5 form the *cAMP signaling pathway* (adjusted-*P* = 1.42 × 10^− 3^). We therefore confirmed expected interactions between genes in a physiological function that is regulated by the alternative splicing of its factors. We further computed the genome-wide co-expression network of *GRIA1* to determine whether the top genes that co-spliced with it also topped in co-expression. We observed a similar but more dramatic pattern than that for *APP* **(**Additional file 1: Figure S12). That is, many more genes highly co-expressed with *GRIA1* than those that highly co-spliced with it. We also observed that no significant pathways were found for the top 20 co-expressed genes. In particular, we found that the co-expression of *GRIA1* and *GRIA3* was much lower ranked (1347 highest correlation with value 0.80) than their co-splicing, suggesting that expression levels are not enough to drive the coregualtion between the genes. This type of specific observations indicate a tight regulation between the genes’ isoforms that can be further investigated.

### ***FGFR2 /CASC4 c***o-splicing is modulated by cancer risk SNPs in breast and prostate

We also asked whether co-splicing correlations could add information to validated transcriptome-wide association studies (GWAS) results. In breast data, we looked at the transcriptome-wide co-splicing correlations of *FGFR2,* whose SNP rs2981582 has been associated with breast cancer [20] and for which numerous splicing forms have been found specifically expressed in breast cancer cells lines [21]. Although correlations were smaller than in previous cases, we found that the top 20 genes that co-spliced with *FGFR2* significantly enriched the pathway *Central carbon metabolism in cancer* (adjusted-*P* = 1.57 × 10^− 2^). We then considered *CASC4,* other gene whose alternative splicing has been linked to breast cancer [40]. We tested whether the co-splicing between the genes was modulated by rs2981582. We used PC derived co-splicing, to test the association of the interaction between the first PC of *FGFR2* and rs2981582 with *the* first PC of *CASC4*, adjusting for covariates (interaction-*β* = 0.10, *P* = 0.017)*.* Interestingly, we replicated the interaction in prostate (interaction-*β* = 0.09, *P* = 0.027), and also found a significant interaction with rs10749415 (interaction-*β* = − 0.14, *P* = 0.038), a SNP in *FGFR2* recently associated with prostate-specific antigen levels [41]. While needing to be confirmed, co-splicing correlations suggest an underlying coregulation of the alternative splicing of *FGFR2* and *CASC4,* modulated by risk SNPs for cancer.

### ***C***o-splicing is ubiquitous across tissues

**Fig. 4:**
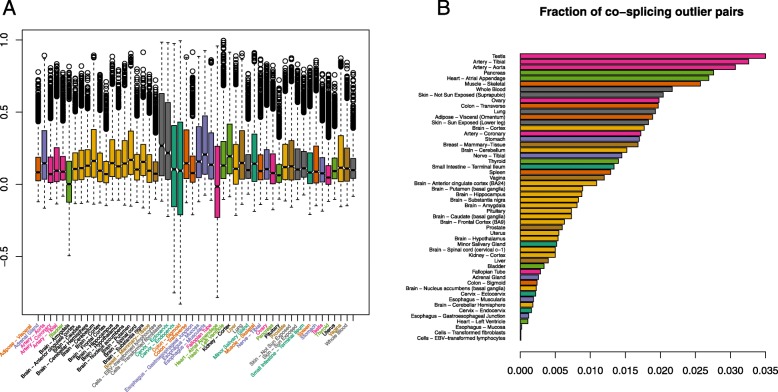
Co-splicing across 52 human tissues. **a** Box plots (box: 3rd, 2nd and 1st quartiles, whiskers: outlier fences of 1.5 interquartile range) for the distributions of co-splicing correlations between all gene-pairs in the genome (17,368 genes) across 52 human tissues, from GTEx. The distributions show numerous outlier pairs with high correlations. **b** Fraction of outlier pairs per tissue shows testis as the tissue with the highest co-splicing rates

We finally analyzed the exon count data from GTEx corresponding to 52 human tissues. For each tissue, we removed genes for which a single exon accounted for 90% of the total gene counts, leaving a total of 17,368. With the aid of a supercomputing facility from the Spanish network for supercomputing (https://www.res.es/), we computed the Mantel co-splicing correlations of all gene pairs across all tissues (coSplicing4GTEx - http://cosplicing.isglobal.org/). We found that co-splicing is ubiquitous across tissues, as numerous gene pairs were observed with high co-splicing with respect to the co-splicing distribution of all possible gene pairs per tissue (Fig. 4a). We computed the fraction of outlier pairs, given by the number of pairs with z-transformed correlations higher than 2.5 times the interquartile range, for each tissue (Fig. 4b). We observed that testis was the tissue with most co-splicing pairs, consistent with previous studies showing this tissue with high rates of alternative splicing events [42].

## Discussion

We showed that coregulation of transcript diversity among genes is a novel predictor of gene function. To support this, we extracted reliable and robust correlates of the transcript diversity of *APP* and we illustrated with three other examples how coregulation of transcript diversity can recover established gene interactions, help to interpret GWAS results and provide new biological insights on their function.

We particularly demonstrated that coregulation of transcript diversity among genes provides new important observations of *APP*’s physiological function and its links to AD. We produced a series of robust and coherent results indicating physiological links between *APP*’s transcript distribution and the proteosomal degradation system, which may be affected during disease. The results are in line with previous knowledge on *APP*’s associations with proteosomal degradation and synaptic signaling [43], adding the important contribution of *APP’*s transcript variety to health and disease, besides transcript volume [10]_._ Our observations particularly support a role played by the regulation of transcript diversity in shaping the gene’s function. In particular, we observed a physiological modulation of the coregulation between *APP* and *UBQLN1* given by UBQ-8i. This result suggests that inconsistencies in the risk of UBQ-8i associated to AD could be derived from the non-accounted disruption of the coregulation between the genes*.* In line with the association between the physiological coregulation of *APP*’s transcription diversity and proteosomal degradation pathways, we also found evidence for the association between *APP* co-splicing and protein aggregation [17, 36, 44]. In particular, we observed that the association of tissue vulnerability to AD was stronger for *APP’s* co-splicing than co-expression. Our result may help to explain why no strong evidence of *APP*’s function is derived from co-expressed genes, as provided by gene function predictors such as GeneMania [45] or STRING [46], in addition to recent transcriptome meta-analyses that have not been able to confirm up-regulation of *APP* in cases with respect to controls [44, 47]. We observe that specific coregulation of transcript diversity explains better the links between the physiological function of *APP* and tissue-vulnerability to disease. Our analyses in particular support new calls for interventions targeting the ubiquitin proteosomal system [48] and underscores the need to study its transcript diversity regulation in association to AD.

We used co-splicing correlations to study the coregulation of transcript diversities among genes. Our estimates are based on exon count distribution data (RNA-seq) which is an indirect measure of isoform mixture [6]. New methods to estimate co-splicing correlations methods should be considered for full-length RNA-sequencing, as it becomes more available. In addition, co-splicing correlations include transcript diversity that could also be derived from alternative transcription at different start and end sites or splicing. The contributions of each of these processes to the correlations need to be further explored because mayor differences in transcript diversity across tissues are given by alternative transcription and not splicing [5]. However, regardless their origin, we show that the coregulation of transcript diversity predicts expected gene correlates. In relation to transcription volume, we observed that changes in transcription quality is likely accompanied with increases in overall gene expression (Fig. 2a), as increments in isoform subgroups are likely to increase the overall expression. Increments in only transcription volume without changes in diversity are also common.

## Conclusions

Co-splicing correlations predict functional correlates of genes. To support this, we extracted reliable and robust correlates of the transcript diversity of *APP* and we illustrated with three other examples how co-splicing correlations can recover established gene interactions, help to interpret GWAS results and provide new biological insights on their function. We also produced a comprehensive and accessible map of co-splicing between genes across numerous human tissues (http://cosplicing.isglobal.org/), showing that the specific co-regulation of genes at isoform level is ubiquitous and can be an important contributor to overall co-expression. Numerous gene function predictors, such as STRING [45], GeneMania [46] or FunCoup [49], amongst many others, currently use multiple sources of evidence that include co-expression but are yet to consider co-splicing. Co-splicing correlations of genes of interest are an important additional source of evidence to predict their function.

## Methods

### Co-splicing correlations between two genes

**Fig. 5:**
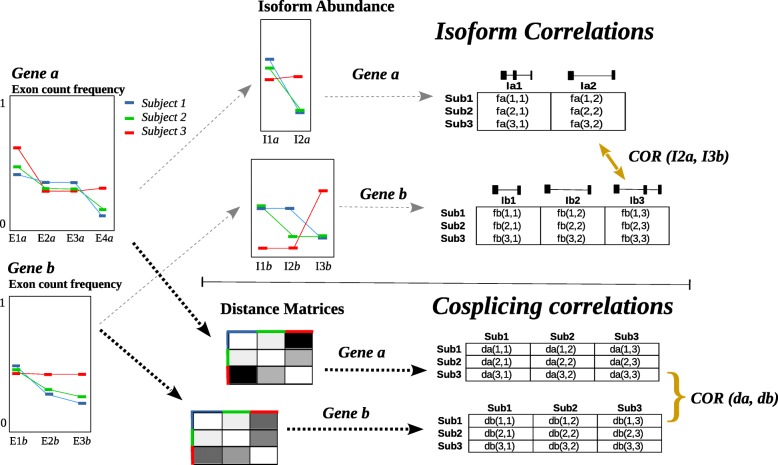
Schematic representation of two pair-wise correlation measures that have been used to derive co-splicing networks (isoform correlations and cosplicing correlations). In the top track, exon count data for two genes is used to infer the isoform abundance for each subject (shown in colors). Gene *a* and gene *b* contain 4 and 3 exons, under 2 and 3 isoforms models, respectively. As, isoform ratios between genes *a* and *b* are obtained, all possible correlations (2*3 = 6) among the isoforms of both genes can be computed. The example illustrates a significant correlation between isform 2 of gene *a* and isoform 3 of gene *b*. In this work, we take the lower track to compute the gene correlations of the co-splicing networks. Here, exon count data is directly used to determine differences between individuals at each gene. No inference of specific isoform models is required and all data is used to determine the distance matrix of individuals. The matrix-based correlations test weather the similarities in the exon count distributions across subjects are kept between genes. The figure illustrates a likely high co-splicing correlation between the genes derived from the fact that, in both genes, the curve of the exon count distributions for subject 3 (red) is substantially different to the curves for subjects 1 and 2 (green and blue). With one single measure the co-splicing correlation between genes, based on distance matrix, informs on the possible coregulation of splicing across all isoforms

The fact that individual exons from different genes can have correlation expression levels, even when there is no correlation between overall expression levels, is due to the coregulation of splicing [6, 7]. Co-splicing networks have been derived in two different ways, each based on a different method to measure the pair-wise coregulation of splicing, see Fig. 5. For instance, Saha et al. have recently used the correlation between individual isoform levels [7]. Here, exon sequencing data is first used to infer the isoform abundance in each gene and then subject correlations between the isoforms across genes are computed. A co-splicing network can then be built form the correlation network based on isform correlations. While this method informs on specific splicing events, it requires isoform inference and numerous correlations to be tested. Using RNA-sequencing (RNA-seq) data, the co-splicing between two genes can also be estimated from the correlation between the exon count distributions of the genes, as proposed by Iancu et al. [6]. This approach is based on distance matrix correlations where a single measure is obtained for each gene-pair. No intermediate isoform inference is required, reducing data processing and likelihood of introducing modeling error. Regarded as a multivariate isoform method, greater detection power can be expected at the expense of interpreting correlations at isoform level. Despite this, the method is adequate for our objective to identify the genes that coregulate their splicing with a gene of interest, and from this perspective, inform on its possible physiological functions.

For matrix based cosplicing cosplicing correlations, the exon count distributions of the genes, per subject, are obtained from the relative frequencies at which the exons of a gene are mapped by reads. The exon count distribution of a gene *a* for subject *i* is therefore defined as the normalized vector

$$ {\mathrm{P}}_{\mathrm{i}}\left(\mathrm{a}\right)=\frac{{\mathrm{a}}_{\mathrm{i}}}{\left|\left|{\mathrm{a}}_{\mathrm{i}}\right|\right|}, $$

of dimension equal to the number of exons in *a*. *a*_*i*_ is vector with Euclidean norm that encodes the counts for each exon of *a* for individual *i*. dThe distance between subjects *i* and *j*, given by their respective exon count distributions of *a*, is computed by the inner product

$$ {\mathrm{d}}_{\mathrm{a}}\left(\mathrm{i},\mathrm{j}\right)=1-{\mathrm{P}}_{\mathrm{i}}\left(\mathrm{a}\right)\circ {\mathrm{P}}_{\mathrm{j}}\left(\mathrm{a}\right). $$

The matrix *d*_*a*_ measures the differences in exon count distributions among individuals, which correlates with the distance matrix of another gene *b* when the genes coregulate their transcript distributions, i.e. their transcript diversities. The overall co-splicing between genes *a* and *b* is computed from the Mantel’s correlation between their distance matrices *Mantel(d*_*a*_*, d*_*b*_*)* [6]. The correlation can be adjusted for transcriptome-wide batch effects using a partial Mantel’s correlation, where the correction matrix is the distance between subjects given by their exon count distributions of the entire genome.

We propose to measure the co-splicing between two genes from the correlation between the first principal components (PC) of the exon count distributions of each gene across subjects. A PC is performed on the exon count distribution of a gene *a* across subjects. We assume that first PC captures the variability in the isoform transcript diversity among subjects. As such, we correlate the first PCs obtained for two genes *a* and *b* using a partial correlation that can be adjusted by covariates. The PC-based co-splicing correlation was used to validate between methods significant co-splicing identified by Mantel’s correlation. In addition the method allows the testing of interactions with genomic variants.

### GTEx data

We downloaded version-6 data from the GTEx project web-site [15]. RNA-seq count data was obtained for 52 different tissues. Pair-ended RNA-seq was performed with Illumina HiSeq 2000 following the TrueSeq RNA protocol. See The GTEx Consortium (2017) and Saha et al. for further details [7]. We were given access to download GTEx genotypes from dbGAP [50] with accession number phs000424.v6.p1. Approximately 1.9 million SNPs were genotyped using whole blood samples with Illumina HumanOmni 2.5 M and 5 M BeadChips. Tissue specific covariates that included transcriptome-wide PC, inferred batch effects and sex were also downloaded from the GTEx web-site.

### BRAINEAC data

We also downloaded the brain expression data of the BRAINEAC project [16] that consists on transcriptomic data of about hundred healthy individuals across 9 different brain tissues. We obtained exon expression data corresponding to Affymetrix Human Exon 1.0 ST array. Gene expression data, from winsorized values, was also downloaded. All data had been previously normalized and corrected for batch effects. See the BRAINEAC consortium for further details.

### Reproducibility of transcriptome-wide co-splicing of *APP* between two independent studies

We studied the co-splicing of *APP* across multiple brain regions, where deposition of amyloid plaques begins. We selected brain regions within the neocortex and the limbic system, which are early affected by amyloid-β deposition, Thal stages 1–3 (23). From the GTEx project, we analyzed data corresponding to the hippocampus (*N* = 94), amygdala (*N* = 72) and hypothalamus (*N* = 96) from the limbic system, and anterior cingulate cortex (*N* = 84), cortex (*N* = 114) and frontal cortex (*N* = 108) from the neocortex. Transcriptome-wide expression data was obtained from the BRAINEAC project, which includes exon-wise expression of 318,197 probes and 26,493 transcripts and genotype data for 134 neurological normal brains in 9 brain regions. Normalized and batch corrected data for Affymetrix 1.0 ST exon array was directly downloaded. From the limbic region, we obtained data for hippocampus (*N* = 130) and, from the neocortex, we downloaded data for frontal cortex (*N* = 134), occipital cortex (*N* = 134) and temporal cortex (*N* = 134). We selected genes between 3 and 40 exon probes, leaving 19,027 transcripts. For this microarray study, we adjusted the exon data by the principal components of the transcriptome-wide distance matrix and use the residuals as exon expression levels. As microarrays are subject-wise normalized, to recover a measure of the exon expression distribution in a gene *a*, we ranked the individuals within each exon and used the subject rankings across *a* to compute *P*_*a*_*(i)* for each subject *i*.

### Pathway enrichment in *APP* co-splicing

We computed, for each study, the enrichment of 277 KEGG pathways in *APP*’s co-splicing correlations, using Bioconductor’s package clusterProfiler. The genes selected for enrichment analysis were those with Mantel correlation *P*-values < 0.05. For each pathway and brain region, we computed the enrichment *P*-values, (Benjamini and Hochberg) adjusted for the number of pathways tested. We additionally adjusted for the number of brain regions and took the minimum adjusted value, across regions, as the *P*-value for the pathway. We look for the pathways significantly enriched in co-splicing in both studies and computed the combined *P*-value between studies. We also tested, using Cohen’s κ, the agreement between studies to declare a pathway significantly enriched in *APP* co-splicing.

### Pathway enrichment in epistases with multiple SNPs in *APP*

We designed a framework to test whether pathways were enriched in epistases with more than one independent SNP of *APP*. The method, an extension of a previous method [38], comprised three steps: (**1**) We first performed genome-wide interaction analyses (GWIA) of late-onset Alzheimer’s disease (LOAD), for uncorrelated SNPs in *APP* (paired-wise R^2^ < 0.2) (see Supplementary Methods). A set of genome-wide additive-by-additive interaction *P*-values was obtained for all uncorrelated *t* SNPs in the gene (*APPsnp*_*i*_, *i = 1...t*), using the likelihood ratio test, χ^2^(1), between the logistic models

$$ \mathrm{y}={\mathrm{snp}}_{\mathrm{j}}\times {\mathrm{APPsnp}}_{\mathrm{i}}+{\mathrm{snp}}_{\mathrm{j}}+{\mathrm{APPsnp}}_{\mathrm{i}}+\mathrm{covariates} $$

and

$$ \mathrm{y}={\mathrm{snp}}_{\mathrm{j}}+{\mathrm{APPsnp}}_{\mathrm{i}}+\mathrm{covariates} $$

where *y* is the case/control status, *snp*_*j*_ encodes the number of variant alleles of the SNP with index *j* in the array, *j* varies over the whole array, and the covariates included the genome-wide principal components and the principal components times *APPsnp*_*i*_. Q-Q plots were created for each GWIA in order to detect and remove *APP*-SNPs with possible false interactions due to unobserved latent variables (Supplementary Tables S1-S3). (2) We then applied an enrichment pathway analysis for all the GWIAs and counted the number of GWIAs, or associated SNPs in *APP*, for which each pathway was found significantly enriched. We used iGSEA4GWAS-v2 on KEGG pathways [51], where we mapped genes to SNPs within 100Kb distance. (3) We tested if the number of GWIAs, for which a pathway was found enriched, was significantly greater than what would be found by chance (Supplementary Methods). False positive rates and statistical power of the method were also assessed (Supplementary Methods).

### LOAD genotype data

We used data of three genome-wide studies of LOAD, all accessible in dbGAP [50]. We selected individuals from European ancestry. The studies are: (1) National Institute of Aging (NIA) study (accession: phs000168.v1.p1) with 587 cases and 289 controls with 590,247 SNPs. (2) GenADA study (phs000219.v1.p1) with a total of 806 cases, 782 controls and 349,252 SNPs. (3) Alzheimer’s Disease Genetics Consortium (ADGC) (phs000372.v1.p1). We kept the first genotyped batch (ADG12) as the second one (ADG3) had limited number of individuals with age at on-set. ADG12 has 2686 cases and 935 controls with 592,652 SNPs. Written informed consent was obtained for all participants at local ethics committee of each study, see dbGap under the accession numbers.

We filtered SNPs and samples by standard quality control measures. We analyzed SNPs with minor allele frequency > 5%, Hardy-Weinberg Equilibrium Z score < 4 and call rate > 80%. At the sample level, genome-wide principal components were calculated with Bioconductors’s snpStats package. Individuals with more than four standard deviations from the mean of the first two components were removed from the analysis. Principal components were recomputed with the selected individuals and the first five components were used in epistasis models as covariates to control for ancestry.

## Additional files

Additional file 1: Supplementary Methods, Supplemental **Figures S1**-**S11.** and **Tables S2**-**S4.** (PDF 968 kb)

Additional file 2: **Table S1.** KEGG Pathways’ enrichment in *APP* co-splicing, co-expression, epistasis and vulnerability index (Delta BI-III) (XLS 58 kb)

Additional file 3: **Table S5.** Pathways with multiple enrichment in *APP* epistasis across three LOAD GWAS. (XLS 58 kb)

## Abbreviations

AD: Alzheimer’s disease
GTEx project: Genotye tissue expression project
GWAS: Genome-wide association studies
GWIA: Genome-wide interaction analysis
KEGG: Kyoto encyclopedia of genes and genomes
LOAD: Late-onset Alzheimer’s disease
SNP: Single nucleotide polymorphism
