# Supplementary material for: When pitch adds to volume: coregulation of transcript diversity predicts gene function
Source: BMC Genomics. 2018 Dec 13;19:926. doi: 10.1186/s12864-018-5263-z (PMC6293560; doi:10.1186/s12864-018-5263-z)
Supplement: Supplementary file 1 — Supplementary Methods, Supplemental Figures S1-S11. and Tables S2-S4. (PDF 968 kb) [file 12864_2018_5263_MOESM1_ESM.pdf]

Supplementary Information

When pitch adds to volume: Coregulation of transcript diversity predicts gene function

Alejadro Cáceres and Juan R. González

Contents

1 Supplementary Methods **2**

1.1 Pathway enrichment in epistases with multiple *APP*-SNPs 2

1.1.1 Significance test for the number of GWIAs in which a pathway is found enriched 2

1.1.2 False positive rate 3

1.1.3 Statistical power 4

2 supplemantary Figures and Tables **6**

2.1 *APP* co-splicing reproducibility between methods 6

2.1.1 supplemantary Figures S1-S2 6

2.2 Q-Q plots of transformed co-splicing correaltions 8

2.2.1 supplemantary Figures S3-S4 8

2.3 UBQ-8i interaction 10

2.3.1 supplemantary Figure S5 10

2.4 Enrichment of *APP* epistasis 11

2.4.1 supplemantary Figures S6-S11 11

2.4.2 supplemantary Tables S2-S4 18

1 Supplementary Methods

1.1 Pathway enrichment in epistases with multiple *APP*-SNPs

We propose a framework to test whether pathways where enriched in epistases with more than one independent SNP of *APP*. The method, an extension of a previous method, is illustrated in the figure below.

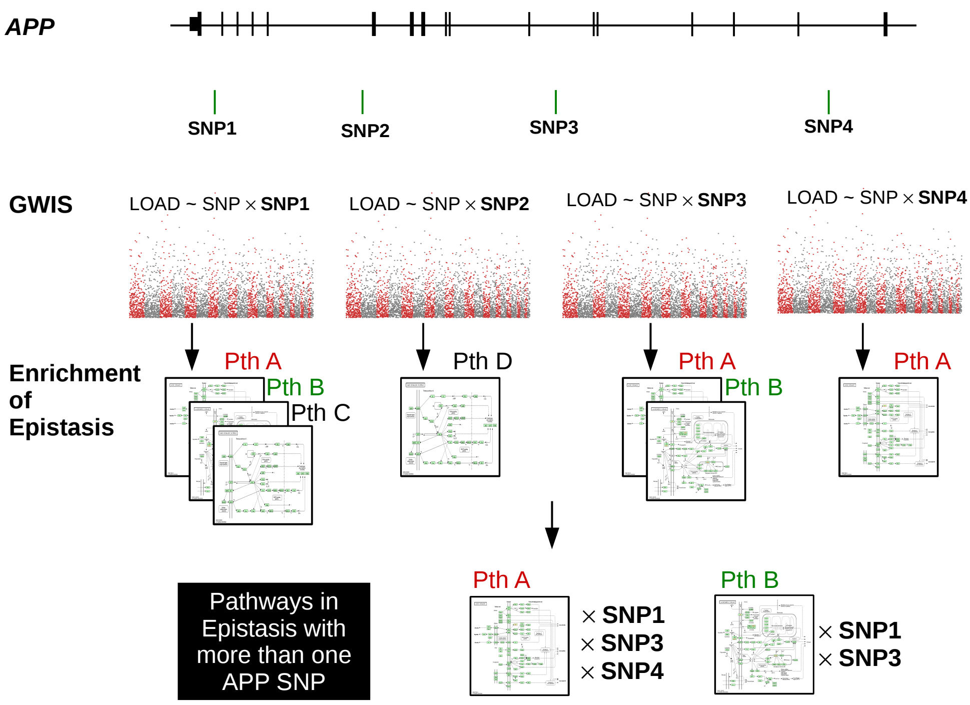

Pathways enriched in epistases with more than one APP-SNP. *The figure is a schematic representation of pathways (Pth A and Pth B) enriched with interactions with more than one SNP within APP, form a total of 4 un-correlated SNPs (t = 4). For instance, pathway A (Pth A at bottom) is found significantly enriched in the genome-wide interactions analysis of SNP1, SNP3 and SNP4 whlie pathway B (Pth B) is enriched in interactions with SNP1 and SNP3.*

1.1.1 Significance test for the number of GWIAs in which a pathway is found enriched

A sampling process was performed for each separate LOAD GWAS, see figure below. The number N of GWIAs in which pathway A (Pth A) is found significant is determined by the number of significant enrichment P-values (reported FDR scores by iGSEA4GWAS-v2) from the total t GWIAs

performed, corresponding to t *APP*-SNPs in the given LOAD GWAS. To determine the statistical significance of N in a given study, K (50) null effect GWIAs were additionally performed based on K SNPs randomly sampled from the genome. K enrichment P-values of epistases with pathway A were then computed $(p_1, ...p_K)$. X subsequent re-samplings with replacement were performed for the number of *APP*-SNPs tested (t) . The number of GWIAs where pathway A was enriched was counted $(n_1, ...n_X)$ for each re-sampling, thus drawing an empirical null distribution to assess the significance of N.

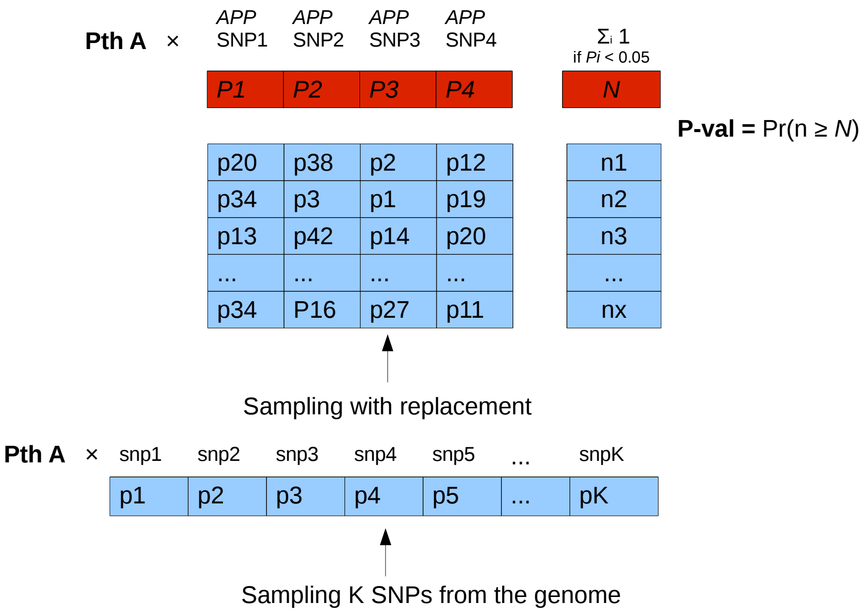

Significance test for the number of GWIAs, APP-SNPs, in which a pathway is found enriched.

1.1.2 False positive rate

We designed sampling and permutation procedures (see figure below) that allowed us to test the likelihood to detect false positive findings, that is the probability that a pathway would be enriched in N GWIAs, from t SNPs randomly selected from the genome. As pathways have different number of genes, of varying lengths, and are differently covered by microarray densities, we tested the overall false positive rate (FPR) for each study separately, as well as for the combination of all three studies. To test FPR in a given study, we first drew a random sample of 50 SNPs from the genome,

for which we did not expect any significant interaction effect. For a study with uncorrelated t SNPs genotyped in *APP*, we re-sampled with replacement 100,000 sets of t SNPs from the previous 50 SNPs. In each re-sample, we counted the number of SNPs that significantly interacted with a given pathway J and calculated a P-value for this number by permuting the pathway levels. A set of 100,000 P-values, corrected for multiple comparisons on the number of pathways tested, was obtained for pathway J. We then computed the pathway's false positive rate (FPRJ), as the number of significant findings at α-level. The total FPR was estimated as the fraction of pathways with FPRJ greater or equal than 5%, as illustrated in the following figure.

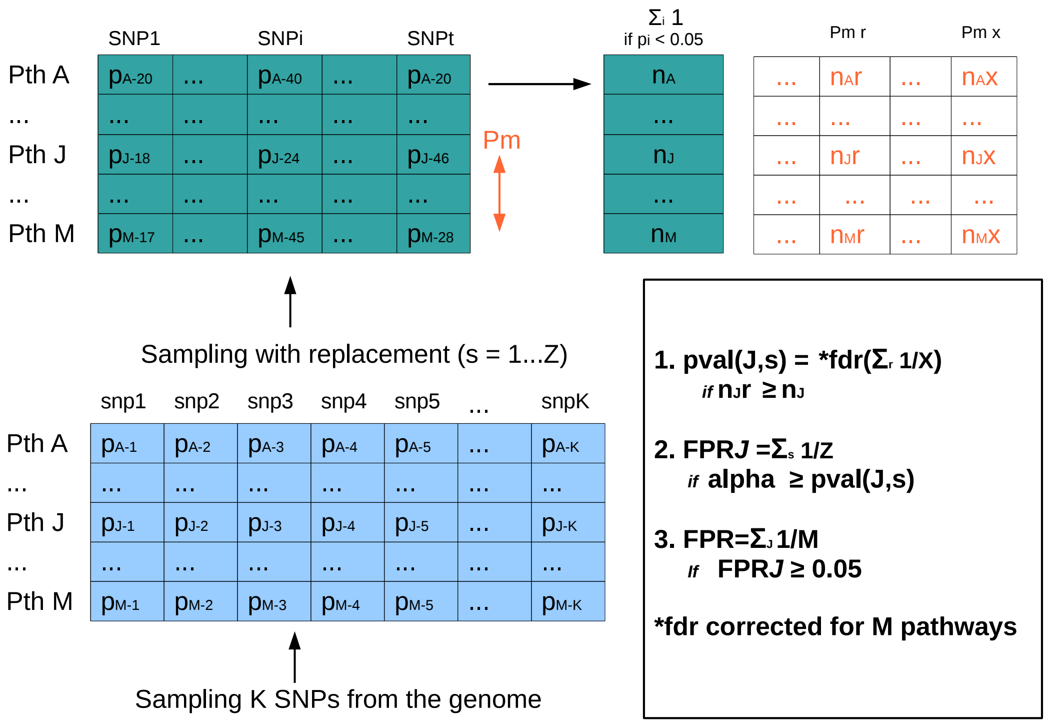

Figure 1: *Scheme of the sampling and permutation procedures to test the fraction of pathways with uncontrolled false positive rate (FPR) in a study were t SNPs are tested for epistatic effects with a set of M pathways.*

1.1.3 Statistical power

We also tested the power to detect a given number of GWIAs in which a pathway is found significantly enriched. Synthetic data were generated using GWAsimulator-2.1 that allows the inclusion

of SNP-SNP interaction terms having different effect sizes [S1]. SNPs were simulated from a reference panel to account for linkage disequilibrium patterns and suitable allele frequencies. Parameters were similar to those used in Caceres *et al.* [37], where sets of 200 enrichment interactions with a given SNP were performed with varying effect sizes and subjects in the study and were embedded in pathways with varying number of genes. Given that, in this study, we were interested in testing the power to find t GWIAs in which a given pathway is enriched, we sampled from the 200 simulations t enrichment P-values and determine the probability to detect all t GWIAs in which the simulated pathway was enriched.

supplemantary References

[S1] Li C, Li M. GWAsimulator: a rapid whole-genome simulation program. Bioinformatics. 2007;24:140-142.

2 supplemantary Figures and Tables

2.1 *APP* co-splicing reproducibility between methods

2.1.1 supplemantary Figures S1-S2

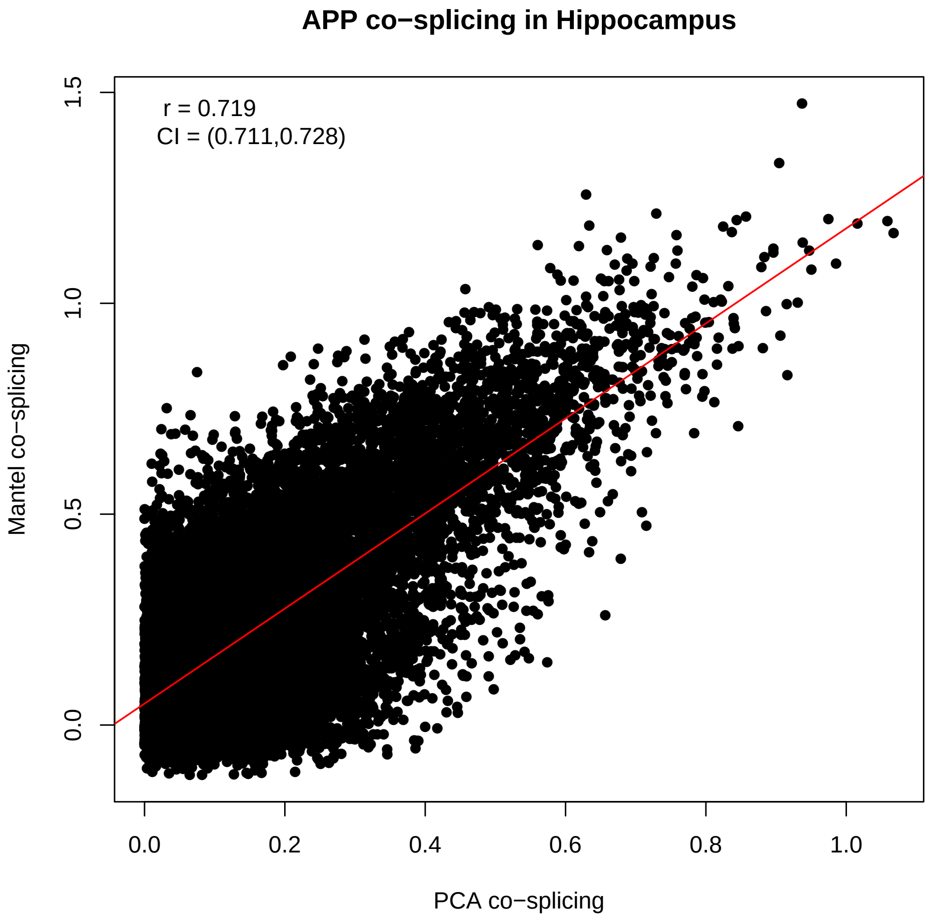

Figure S1. Reproducibility of transcriptome-wide *APP* co-splicing. Reproducibility between Mantel and PCA methods in the GTEx study. Each point represents a gene for which the z-transfomred Mantel's (y axis) and PCA (x axis) co-splicing correlations have been computed.

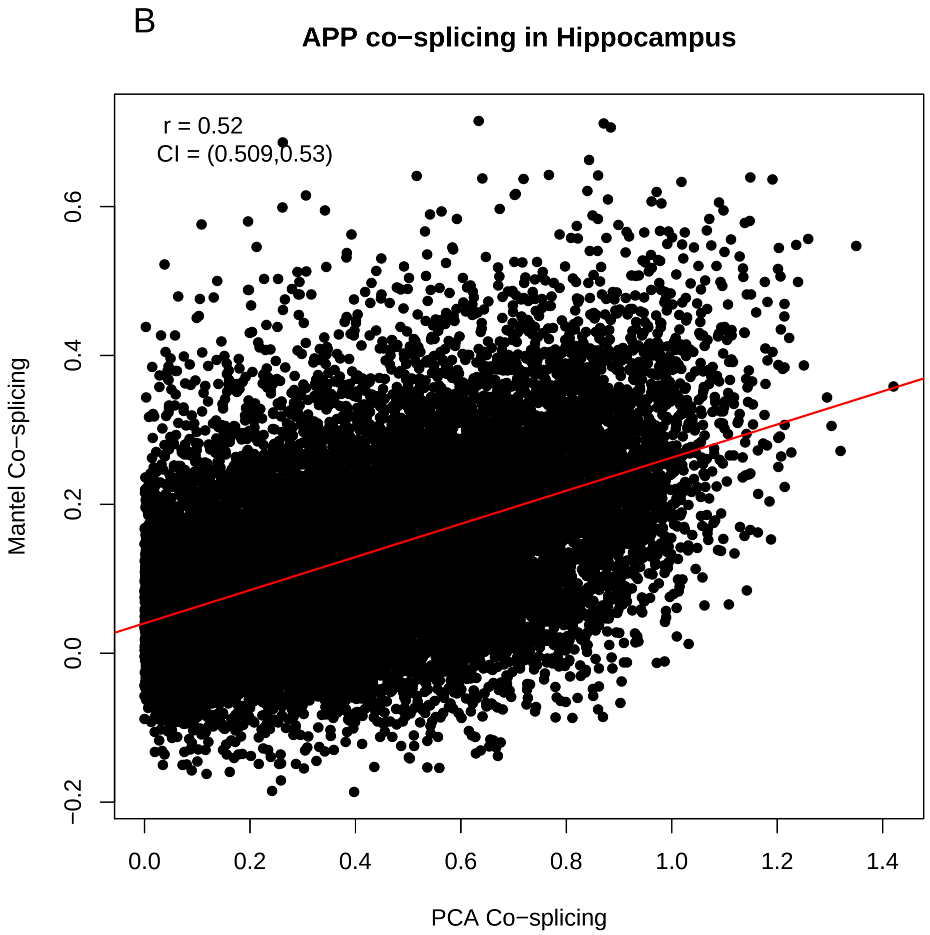

Figure S2. Reproducibility of transcriptome-wide *APP* co-splicing. Reproducibility between Mantel and PCA methods in the BRAINEAC study. Each point represents a gene for which the z-transfomred Mantel's (y axis) and PCA (x axis) co-splicing correlations have been computed.

2.2 Q-Q plots of transformed co-splicing correaltions

2.2.1 supplemantary Figures S3-S4

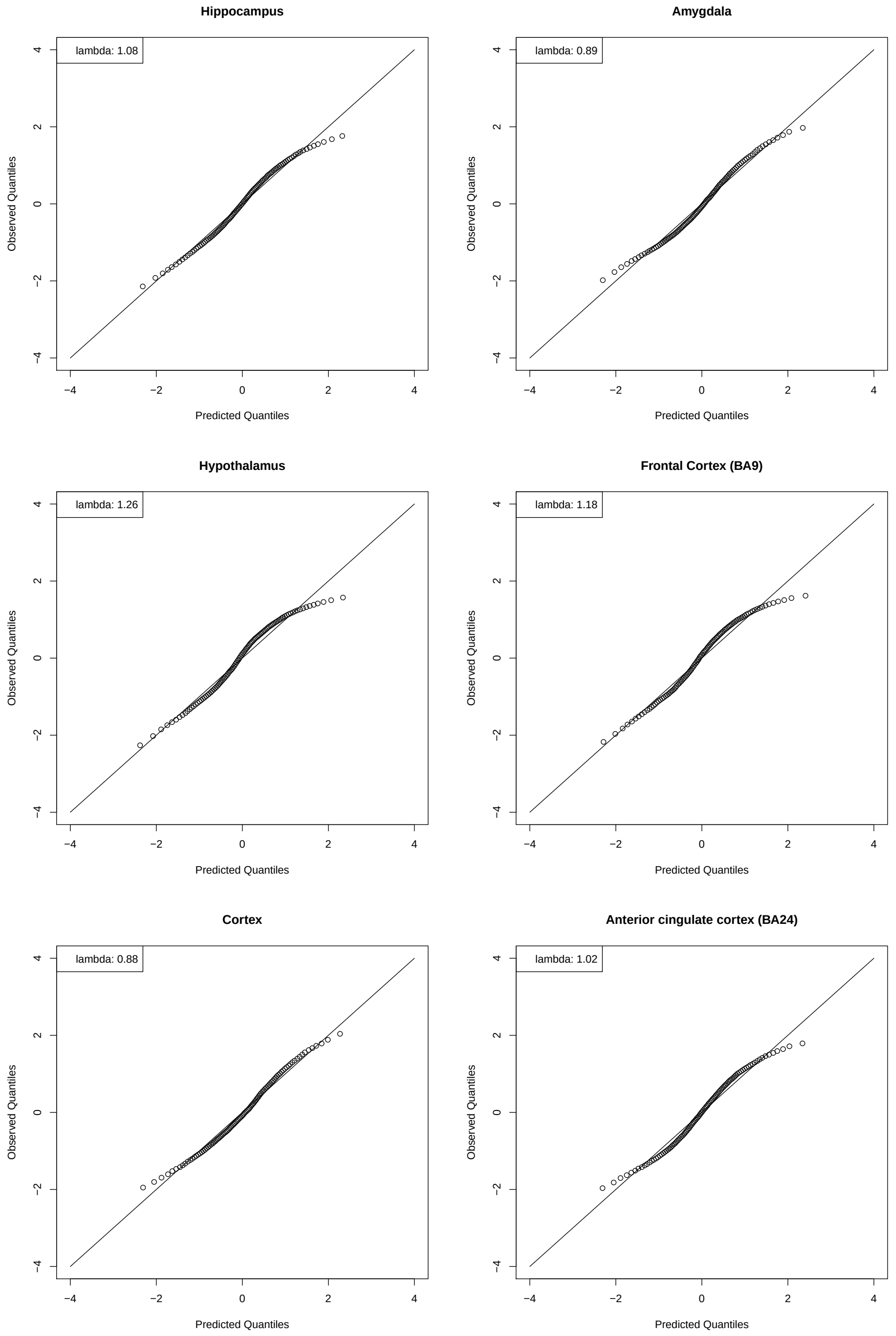

Figure S3. QQ plots of transformed *APP* co-splicing correlations across 6 different tissues in the GTEx study.

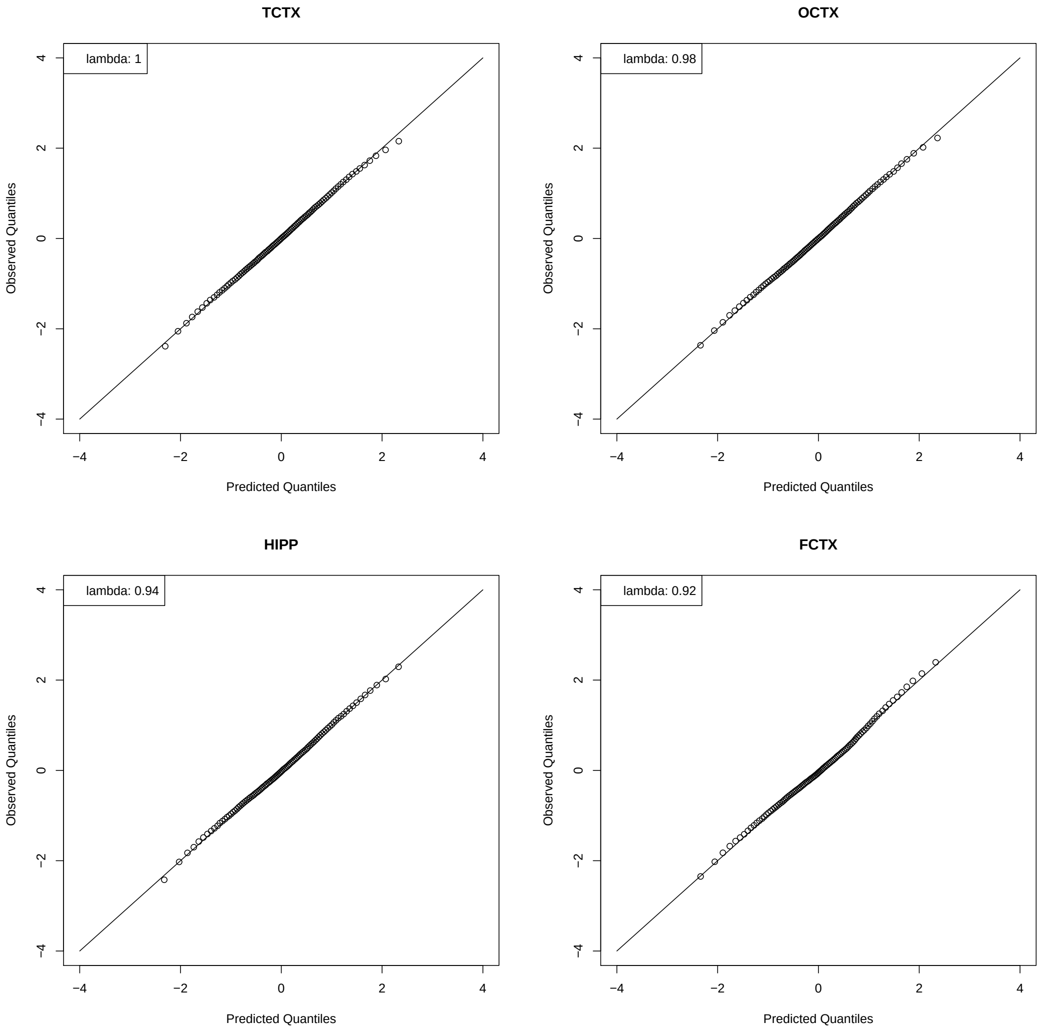

Figure S4. QQ plots of transformed *APP* co-splicing correlations across 6 different tissues in the BRAINEAC study. TCTX: temporal cortex, OCTX: occipital cortex, HIPP: Hipoccampus, FCTX: Frontal Cortex

2.3 UBQ-8i interaction

2.3.1 supplemantary Figure S5

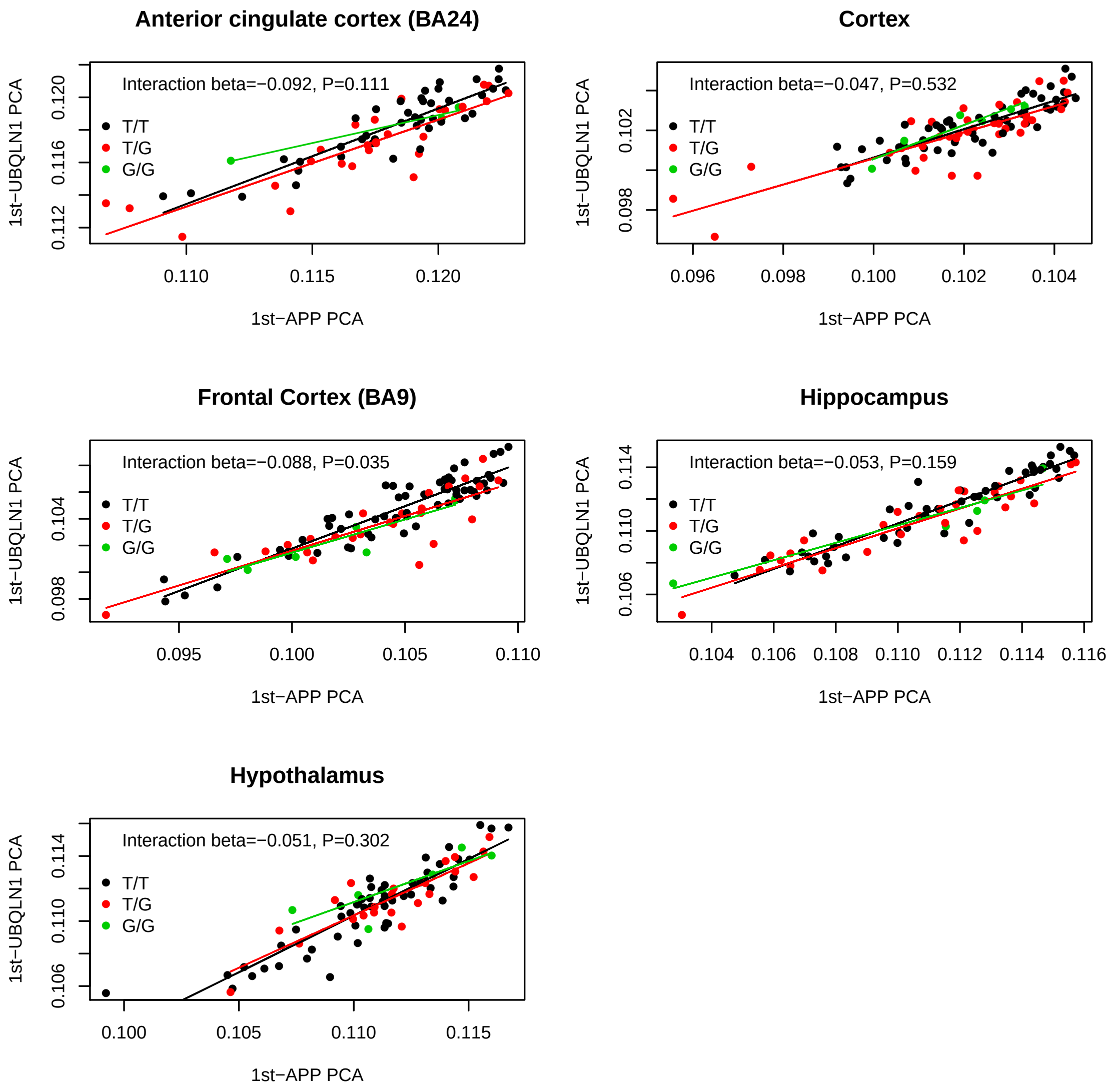

Figure S5. UBQ-8i modulates the co-splicing between *APP* and *UBQLN1*. Test of association for the interaction between UBQ-8i genotypes and 1st *APP* PCA component associated with the 1st *UBQLN1* PCA component, across different sittues and after adjusting for genome-wide covariates.

2.4 Enrichment of *APP* epistasis

2.4.1 supplemantary Figures S6-S11

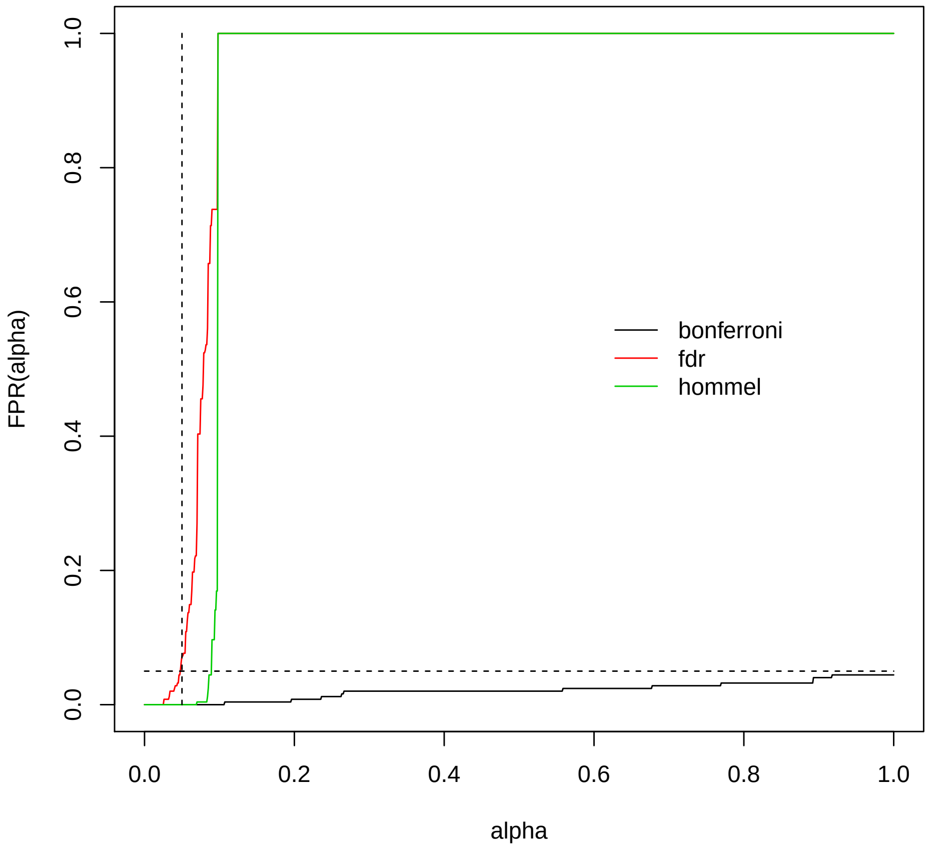

Figure S6. False positive rate FPR(alpha) for ADG study. FPR was computed following the scheme explained in Methods for the ADG study. Three corrections for multiple comparisons were tested: Bonferroni, FDR and Hommel. The amount of pathways with false positive rates greater than 5% was kept under control when the detection threshold alpha is set at 0.05. Bonferroni correction is clearly restrictive.

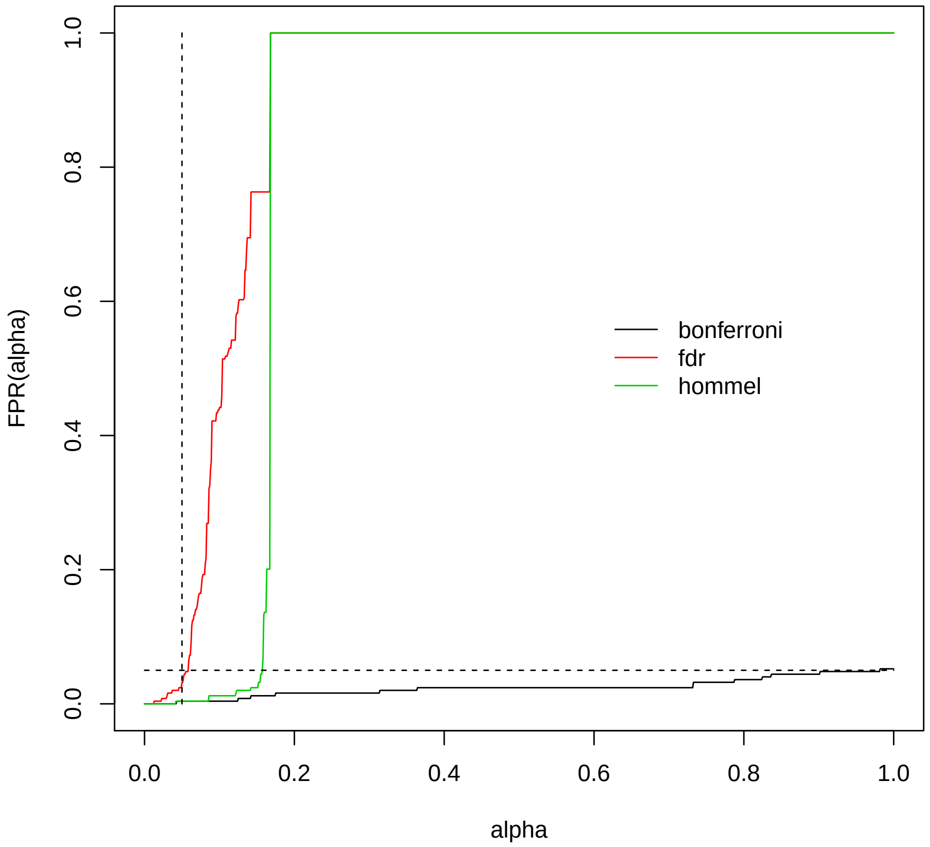

Figure S7. False positive rate FPR for NIA study. FPR was computed following the scheme explained in Methods for the NIA study. Three corrections for multiple comparisons were tested: Bonferroni, FDR and Hommel. The amount of pathways with false positive rates greater than 5% was kept under control when the detection threshold alpha is set at 0.05.

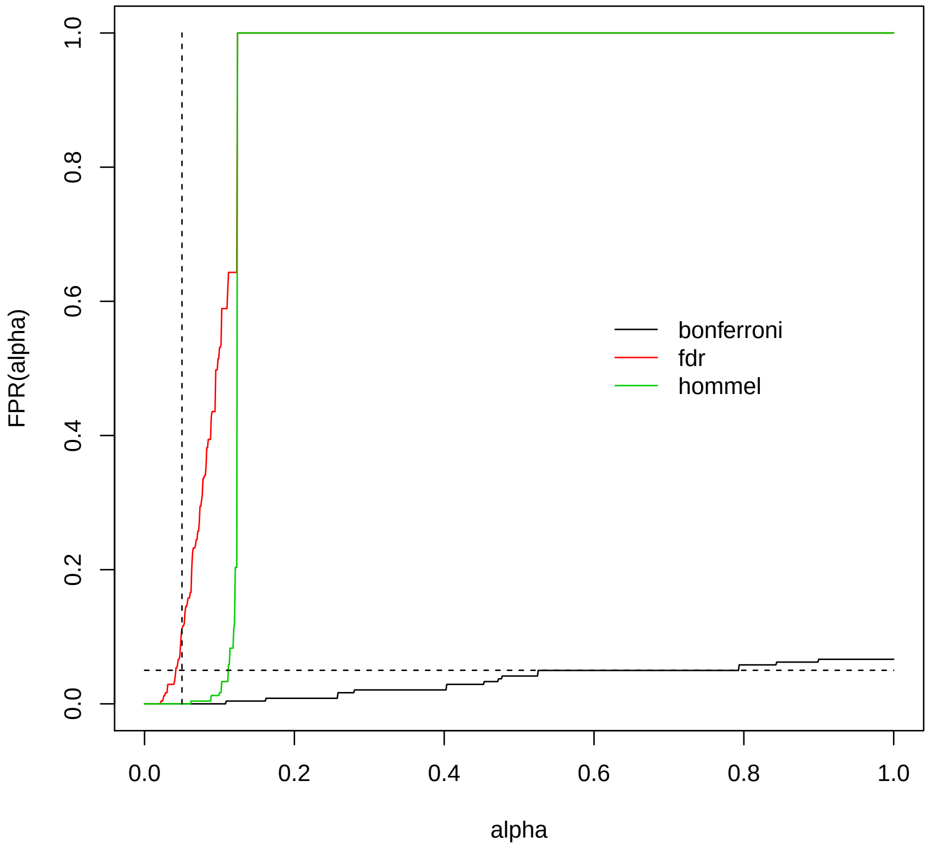

Figure S8. False positive rate FPR(alpha) for GENADA study. FPR was computed following the scheme explained in Methods for the GENADA study. Three corrections for multiple comparisons were tested: Bonferroni, FDR and Hommel. The amount of pathways with false positive rates greater than 5% was kept under control when the detection threshold alpha is set at 0.05. However, in this case, FDR correction was not sufficient.

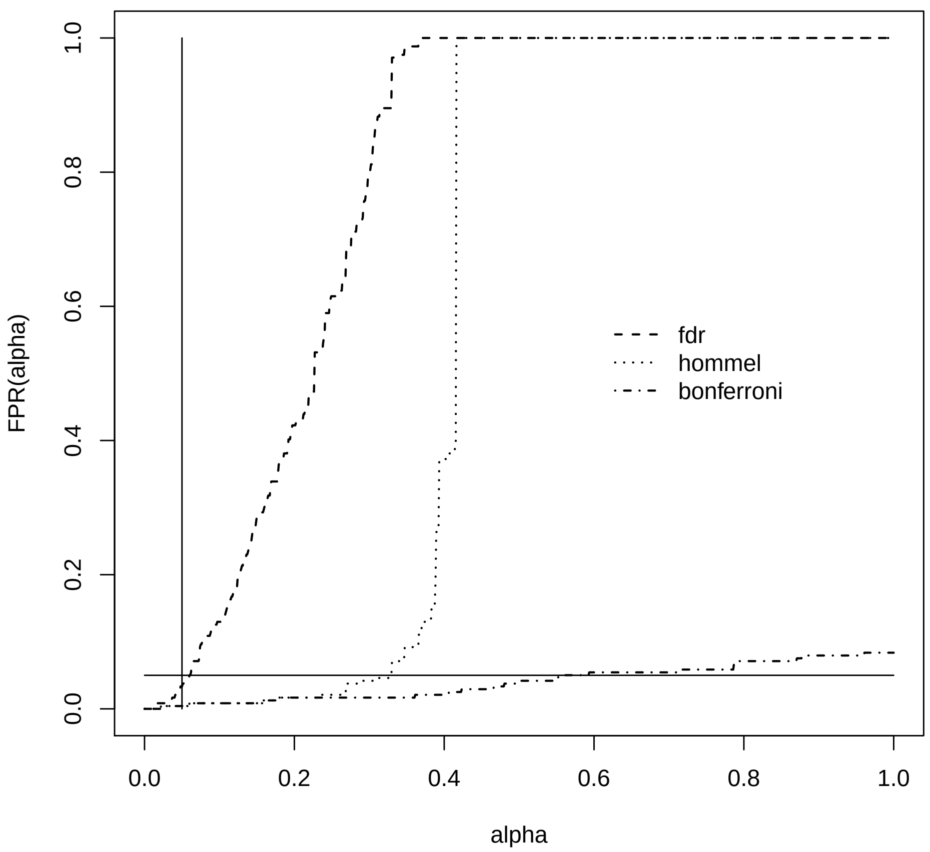

Figure S9. False positive rate FPR for combined analysis. FPR was computed following the scheme explained in Methods for the combined analysis of all three studies. Three corrections for multiple comparisons were tested: Bonferroni, FDR and Hommel. The amount of pathways with false positive rates greater than 5% was kept under control when the detection threshold α is set at 0.05. Bonferroni correction is clearly restrictive.

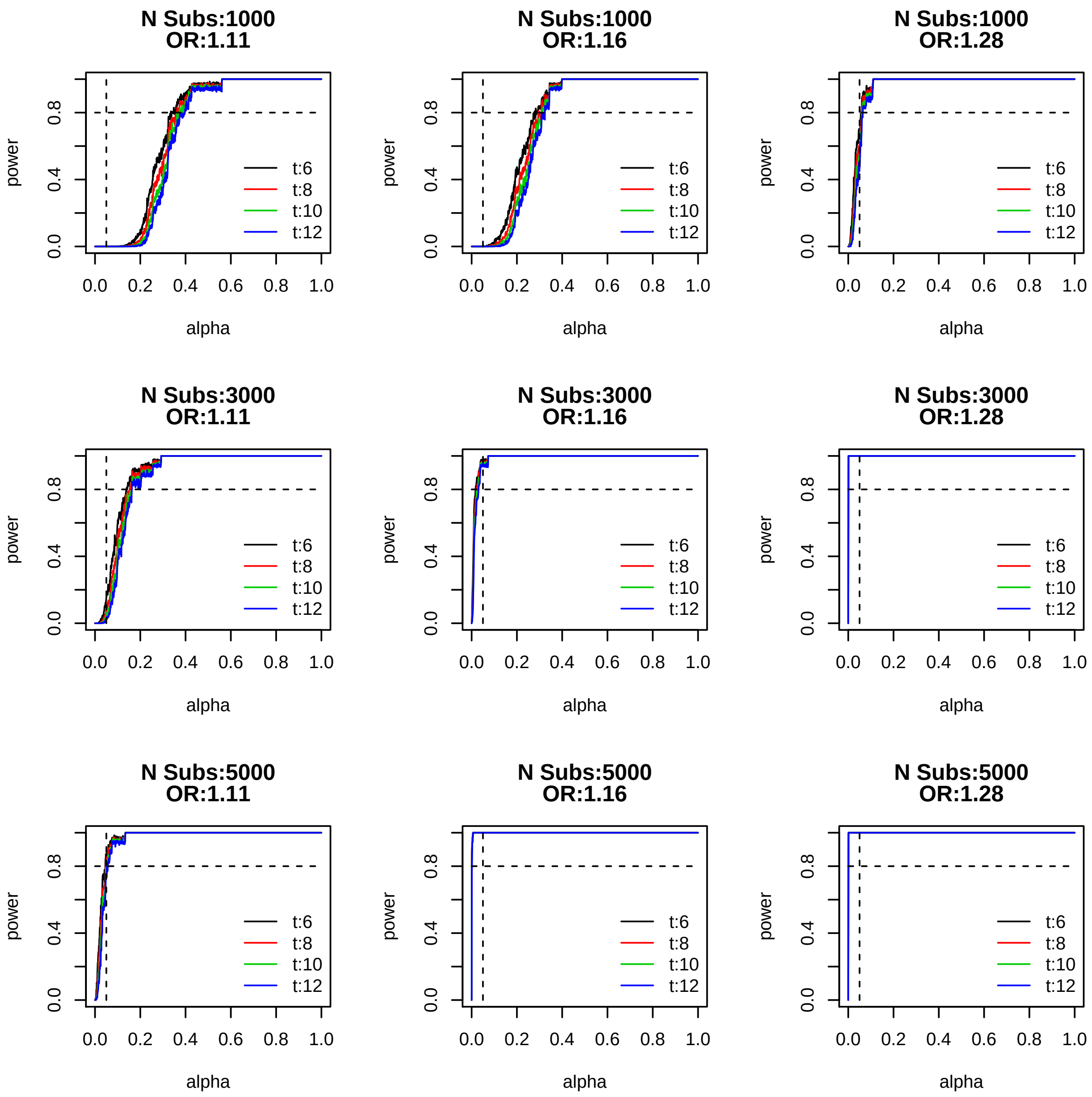

Figure S10. Power calculations using GWAsimulatior2-1. Simulations were preformed for a pathway with 106 genes, embedded in the genome, that was enriched in interactions with varying number of SNPs (t) at varying size effects (odds ratio OR) and in studies with different number of subjects (N Subs).

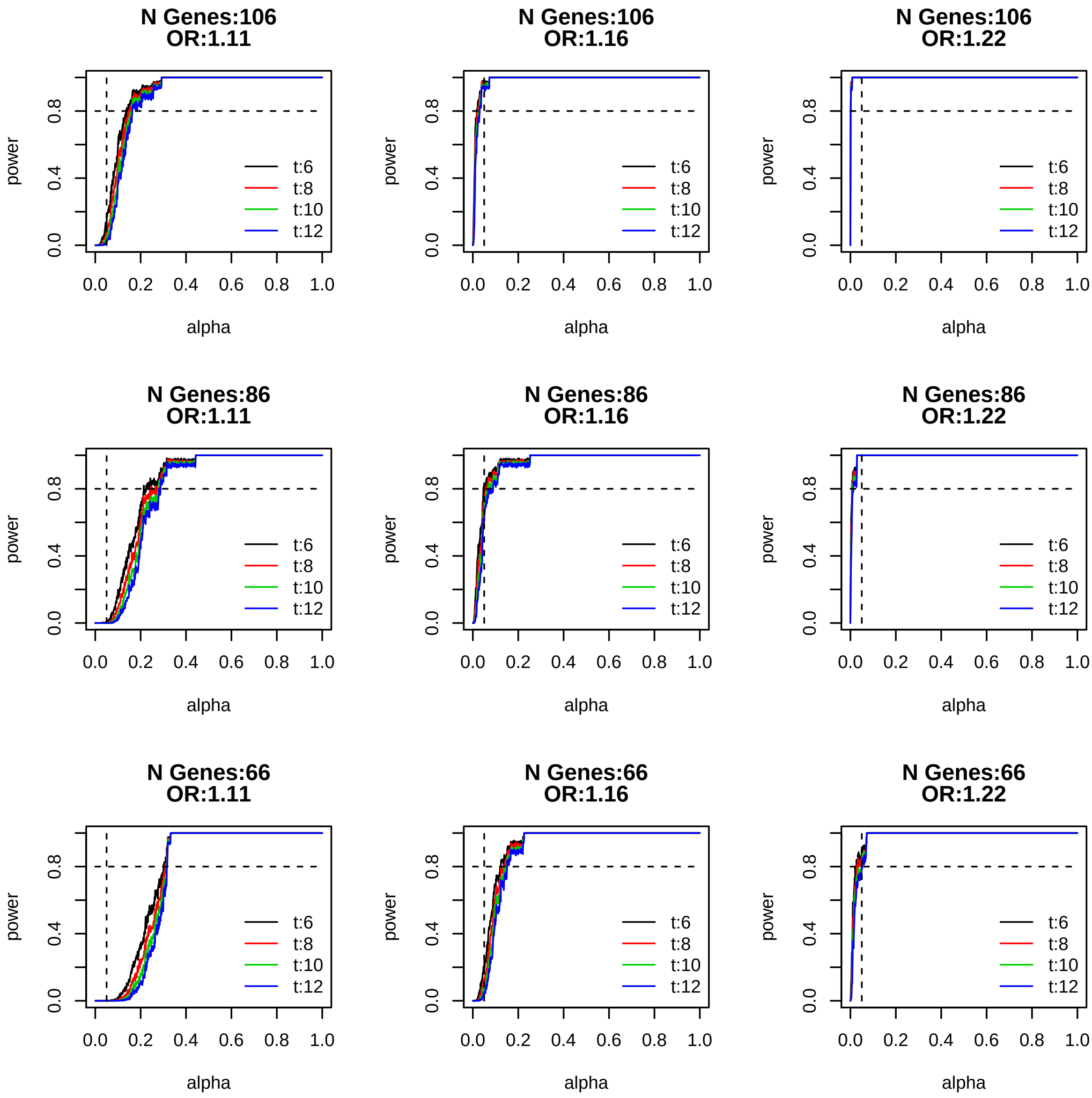

Figure S11. Power calculations using GWAsimulatior2-1. Simulations were preformed for a study of 3000 individuals for three pathways with varying number of genes (N Genes), embedded in the genome, that were enriched in interactions with varying number of SNPs (t) at varying intensities (odds ratio OR).

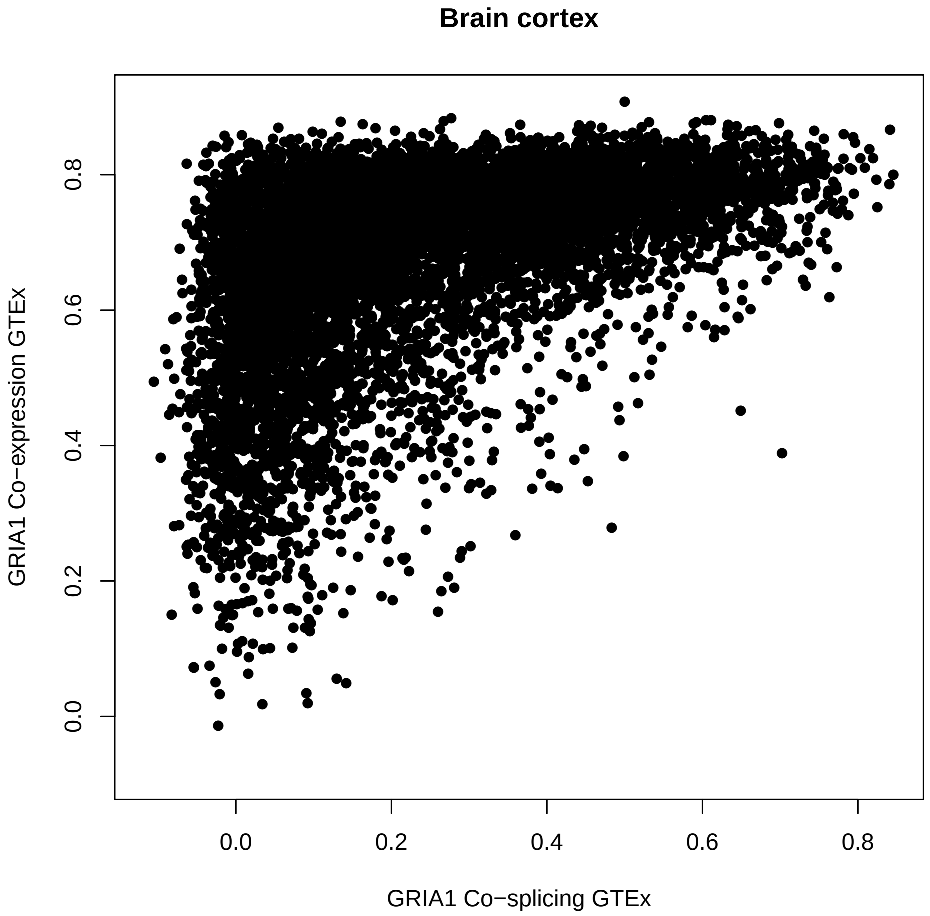

Fig S12. Correlation between transcriptome-wide *GRIA1* co-expression and co-splicing correlations. Each point is a gene for which its co-expression and co-splicing correlations (z-transformed) with *GRIA*1 were computed. A high correlation was found for brain cortex data of GTEx, where high co-splicing is a clear subset of high co-expression.

2.4.2 supplemantary Tables S2-S4

SNP name	Allele 1	Allele 2	Call rate	RAF	z.HWE	QQlambda	selected
rs3787620	C	A	1	0.89	-0.33	1.13	FALSE
rs373521	A	C	1	0.63	-0.71	1.02	TRUE
rs380417	T	C	1	0.76	-2.82	1.02	TRUE
rs1783016	T	C	1	0.75	0.01	1.06	TRUE
rs2234983	A	G	1	0.83	-0.75	1.06	TRUE
rs2014146	G	A	1	0.61	-0.66	1.01	TRUE
rs768039	T	C	1	0.7	-0.2	1.04	TRUE
rs2830028	T	G	0.99	0.75	-0.88	1.03	TRUE
rs3991	T	G	1	0.78	-0.34	1.03	TRUE
rs7283136	C	T	1	0.94	-1.45	1.06	TRUE
rs2830040	T	C	1	0.94	-0.16	1.06	TRUE

Table S2. 12 SNPs within *APP* for ADG study and with pair-wise LD $R^2 < 0.2$. A GWAIs was run for each SNP and the Q-Q inflation parameter (λ) computed. We selected 8 SNPs whose $0.9 < \lambda < 1.1$, for enrichment analysis. Allele 1: Reference allele, Allele 2: alternative allele, RAF: reference allele frequency, z.HWE: z-score for Hardy Weinberg Equilibrium test, selected: whether selected for enrichment analysis.

SNP name	Allele 1	Allele 2	Call rate	RAF	z.HWE	QQlambda	selected
rs3787620	C	A	1	0.89	-0.33	1.13	FALSE
rs373521	A	C	1	0.63	-0.71	1.02	TRUE
rs380417	T	C	1	0.76	-2.82	1.02	TRUE
rs1783016	T	C	1	0.75	0.01	1.06	TRUE
rs2234983	A	G	1	0.83	-0.75	1.06	TRUE
rs2014146	G	A	1	0.61	-0.66	1.01	TRUE
rs768039	T	C	1	0.7	-0.2	1.04	TRUE
rs2830028	T	G	0.99	0.75	-0.88	1.03	TRUE
rs3991	T	G	1	0.78	-0.34	1.03	TRUE
rs7283136	C	T	1	0.94	-1.45	1.06	TRUE
rs2830040	T	C	1	0.94	-0.16	1.06	TRUE

Table S3. 11 SNPs within *APP* for NIA study and with pair-wise LD $R^2 < 0.2$. A GWAIs was run for each SNP and the Q-Q inflation parameter (λ) computed. We selected 10 SNPs whose $0.9 < \lambda < 1.1$, for enrichment analysis. Allele 1: Reference allele, Allele 2: alternative allele, RAF: reference allele frequency, z.HWE: z-score for Hardy Weinberg Equilibrium test, selection: whether selected for enrichment analysis.

SNP name	Allele 1	Allele 2	Call rate	RAF	z.HWE	QQlambda	selected
rs1701003	C	G	1	0.6	1.95	1	TRUE
rs454017	T	G	1	0.81	-0.08	0.98	TRUE
rs1783016	T	C	1	0.75	-0.07	0.99	TRUE
rs216763	A	T	1	0.92	-0.73	0.97	TRUE
rs216772	A	T	1	0.94	0.31	0.92	TRUE
rs11087985	C	A	1	0.64	0.67	0.95	TRUE
rs2830031	C	T	1	0.69	-1	1	TRUE
rs9980729	C	G	1	0.94	-1.43	0.82	FALSE
rs9981258	A	C	1	0.73	-0.18	0.95	TRUE
rs2830053	T	C	1	0.89	0.57	0.94	TRUE

Table S4. 10 SNPs within *APP* for GENEADA study and with pair-wise LD $R^2 < 0.2$. GWAIs was run for each SNP and the Q-Q inflation parameter (λ) computed. We selected 9 SNPs whose $0.9 < \lambda < 1.1$, for enrichment analysis. Allele 1: Reference allele, Allele 2: alternative allele, RAF: reference allele frequency, z.HWE: z-score for Hardy Weinberg Equilibrium test, selection: whether selected for enrichment analysis.
